# Double-crowned 2D semiconductor nanoplatelets with bicolor power-tunable emission

**DOI:** 10.1038/s41467-022-32713-2

**Published:** 2022-08-30

**Authors:** Corentin Dabard, Victor Guilloux, Charlie Gréboval, Hong Po, Lina Makke, Ningyuan Fu, Xiang Zhen Xu, Mathieu G. Silly, Gilles Patriarche, Emmanuel Lhuillier, Thierry Barisien, Juan I. Climente, Benjamin T. Diroll, Sandrine Ithurria

**Affiliations:** 1 Laboratoire de Physique et d’Etude des Matériaux, ESPCI-Paris, PSL Research University, Sorbonne Université Univ Paris 06, CNRS UMR 8213, 10 rue Vauquelin, 75005 Paris, France; 2 Sorbonne Université, CNRS, Institut des NanoSciences de Paris, INSP, F-75005 Paris, France; 3 Synchrotron SOLEIL, L’Orme des Merisiers, Départementale 128, 91190 Saint-Aubin, France; 4 Centre de Nanosciences et de Nanotechnologies, CNRS, Université Paris-Saclay, C2N, Palaiseau, 2110 France; 5 Departament de Quimica Fisica i Analitica, Universitat Jaume I, E-12080 Castello de la Plana, Spain; 6 Center for Nanoscale Materials, Argonne National Laboratory, Lemont, IL 60439 US

**Keywords:** Quantum dots, Quantum dots

## Abstract

Nanocrystals (NCs) are now established building blocks for optoelectronics and their use as down converters for large gamut displays has been their first mass market. NC integration relies on a combination of green and red NCs into a blend, which rises post-growth formulation issues. A careful engineering of the NCs may enable dual emissions from a single NC population which violates Kasha’s rule, which stipulates that emission should occur at the band edge. Thus, in addition to an attentive control of band alignment to obtain green and red signals, non-radiative decay paths also have to be carefully slowed down to enable emission away from the ground state. Here, we demonstrate that core/crown/crown 2D nanoplatelets (NPLs), made of CdSe/CdTe/CdSe, can combine a large volume and a type-II band alignment enabling simultaneously red and narrow green emissions. Moreover, we demonstrate that the ratio of the two emissions can be tuned by the incident power, which results in a saturation of the red emission due to non-radiative Auger recombination that affects this emission much stronger than the green one. Finally, we also show that dual-color, power tunable, emission can be obtained through an electrical excitation.

## Abstract (web summary)

Nanocrystals are desirable light sources for advanced display technologies. Here, the authors report on double-crowned 2D semiconductor nanoplatelets as light downconverters that offer both green and red emissions to achieve a wide color gamut.

## Introduction

Semiconductor nanocrystals (NCs) are nanoparticles with size-tunable optical features thanks to quantum confinement. Beyond the ease to induce a spectral shift, NCs also offer a narrow photoluminescence (PL) signal resulting from low ensemble polydispersity. This property is of utmost interest for the design of down converters for displays. Currently, a quantum dot display relies on a blue light-emitting diode (LED) based on InGaN quantum wells used to excite two populations of NCs emitting in the green and the red. The combination of these three colors is used to generate white light that is later filtered through a liquid crystal filter to generate red, green, and blue pixels. The human eye is most sensitive to green light. For this reason, in this spectral range, a limited change of the spectral linewidth drastically affects the color gamut (i.e., the color palette), while for blue and red the linewidth appears less critical. Thus, designing NCs with narrow green emission is essential to achieve high-quality displays.

**Fig. 1:**
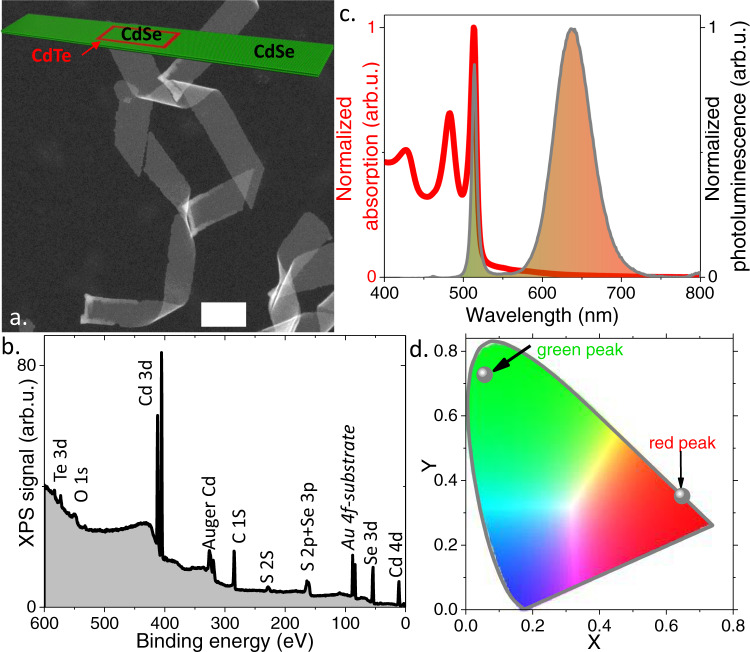
Structural and spectral properties of CdSe/CdTe/CdSe core/crown/crown NPLs. **a** HAADF-STEM image of the CdSe/CdTe/CdSe core/crown/crown NPLs. The scale bar is 100 nm. The top inset is a schematic of an unfolded NPL. **b** X-ray photoemission overview spectrum for the same NPLs. **c** Absorption and photoluminescence spectra of the NPLs. Feature at 460 nm is due to residue of 3.5 ML CdSe NPL. **d** Chromaticity diagram and positions associated with the green and red features.

Green emissions can easily be obtained from III-V (using InP) and II-VI (using CdSe) semiconductors. However, using spherical particles, the PL linewidth of CdSe remains above 25 nm and even higher using InP. CdSe 2D nanoplatelets^1–3^ (NPLs), thanks to a specific growth mechanism^4–6^, offer the narrowest PL linewidth among NCs. The 2D growth enables atomic control of the thickness, its only confined direction. As a result, the PL linewidth is limited by homogeneous broadening^7^. When they are grown with a thickness of 4.5 monolayers (MLs) (i,e., four planes of Se sandwiched by five planes of cadmium), their PL emission presents a maximum at ~510 nm at room temperature, close to the optimal green wavelength for displays, see Fig. 1d.

As stated before, the current NC display technology relies on the blend of two populations of NCs embedded into a transparent matrix whose role is to protect the NCs from oxidation, and also to extract heat generated by sub unity PL quantum yield. To maintain long-term stability, this matrix is typically covalently bound to the NC surface. Integration of two NC populations (i.e., one green and one red) makes this chemical coupling more complex. It is thus of utmost interest to design a single NC that (i) combines both emissions, while (ii) presenting a narrow green PL linewidth. In the past, several works have reported bicolor emission from NCs^8–12^, however, none of these previous works combine the two properties simultaneously. These two constrains require the design of a system that does not obey Kasha’s rule which states that the emission should occur through the lowest excited state due to fast thermalization of the hot carriers. To do so, the non-radiative decay paths and the carrier cooling need to be drastically slowed down. NPLs, with their large volume, appear as promising candidates^13,14^.

NPLs offer a nice playground for quantum engineering of excitonic transitions through heterostructure design, especially with CdSe core/crown geometry^12,13^ where a second semiconductor is laterally extended around the CdSe core without modification of the thickness. The initial confinement of the NPL is maintained and can thus be used to generate a narrow green emission. Few reports mention bicolor emission from NPLs. Dufour et al. designed a CdSe/CdSeTe core/crown heterostructure presenting two distinct emissions^9^, however the two orange and red emissions were spectrally overlapping. Khan *et al*. designed a core/barrier/crown NPL^15^ as a photon up converter with both green and red emissions. However, the red light was always largely prevailing over the green emission. This latter work suggests that in addition to the combination of a red and a narrow green emission, the two emissions need to be relatively close in magnitude and possibly tunable. Here, we design a single NPL emitter combining the narrow green emission together with a red emission, with tunable relative magnitude of the two peaks. We demonstrate that the introduction of a small CdTe belt in a large CdSe NPL forming a CdSe core/CdTe crown/CdSe crown heterostructure fulfills all the targeted objectives. We start by discussing the geometry of such NPLs and present the optical transitions involved. We then investigate, experimentally and theoretically, the power-dependence of the spectrum. Finally, we demonstrate that bicolor, power-tunable electroluminescence can also be obtained from this core/crown/crown NPL species.

## Discussion and results

### Design of bicolor-emitting nanoplatelets

To achieve a wide color gamut, it is critical to use a narrow green emitter. Thus, our general design relies on the use of the green emission of 4.5 MLs thick CdSe NPL with an emission peak at 510 nm, see supplementary note 1. We then design a heterostructure around this building block. Core/crown heterostructures offer the possibility to spatially integrate uncoupled materials in the same object. Several strategies have been tested to generate the red emission in addition to the green one, (see supplementary note 2), including core/crown/crown with an external alloyed crown (CdSe/CdS/CdSeTe) and core/crown/crown/crown (CdSe/CdS/CdSe/CdTe). But none of them were fully satisfying with an intense orange emission for the first one and a poor green to red emission ratio for the second one. However, the type-II band alignment associated with the CdSe/CdTe interface enables a well-suited deep red emission. Its magnitude is driven by the perimeter of this interface between the two semiconductors. Thus, it is required to design a heterostructure where this interface is as small as possible and where the CdSe area is sufficiently extended to favor the green narrow emission. Hence we synthesized CdSe/CdTe/CdSe core/crown/crown NPLs, in other words a CdSe crown is added to the well-established CdSe/CdTe core/crown NPLs^16–20^, see supplementary note 3. This structure combines the large volume of the NPLs^13,14^ and the type-II band alignment of the CdSe/CdTe interface^21,22^ which are both favorable to slow the carrier cooling and design a structure overruling Kasha’s rule.

To grow such heterostructures, we start with 4.5 ML CdSe NPL cores with 7 × 33 nm^2^ large, laterally extended with a very narrow CdTe crown of a few nm in width. The second external CdSe crown is directly grown with an extension of around 60 nm. The final amount of Te is low and generally around the detection threshold (≈1%, see supplementary note 5) for energy-dispersive X-ray spectroscopy measurements. Nevertheless, by conducting selective etching of the NPLs while exposing them to oleylamine, we can reveal the localization of the initial core in the final NPLs and this is generally observed shifted from the NPL center as previously reported for dot in rods structure, see supplementary note 5. The presence of Te is confirmed by X-ray photoemission, see Fig. 1b and supplementary note 7. A schematic of a final NPL is given as inset of Fig. 1a, while transmission electron microscopy reveals that NPLs tend to fold. The NPL bending does not have a significant effect on the electronic structure, as confirmed by the fact that the band-edge feature of CdSe appears at the same wavelength as for core-only CdSe NPLs (which have no bending). This is because curvature radius is large (tens of nm).

Due to the low Te content, the overall absorption spectrum of the CdSe/CdTe/CdSe core/crown/crown NPLs is, as expected, very similar to the one observed for CdSe NPLs only. We nevertheless observe at wavelengths longer than those of CdSe core some residual absorption. It includes three contributions: (i) the band edge of 4.5 ML CdTe NPLs at 550 nm, (ii) the absorption of the indirect exciton around 650 nm even though the associated cross-section is generally low^23,24^, and finally (iii) the light scattering at low energy due to the large lateral extension of NPLs.

The PL spectrum with lamp excitation (i.e. even under low excitation power) displays two spectrally disjoint features. The one at 510 nm corresponds to the CdSe emission, where the CIE (Commission Internationale de l’Éclairage) coordinates associated with this peak are (0.06; 0.73) and the second with a maximum between 600 and 650 nm (CIE coordinate (0.65;0.35)) corresponds to the CdSe/CdTe interface emission. The geometrical factors of the NPLs can be used to tune the spectrum and the relative magnitude of the two peaks. The CdTe crown size can induce a shift of the red emission, see supplementary note 3, due to lateral confinement in the crown. On the other hand, the lateral extension of the CdSe crown drives the relative magnitude of the red and green signals. The larger the crown, the higher the green to red emission ratio, see supplementary note 4.

### Photophysics of bicolor emission

**Fig. 2:**
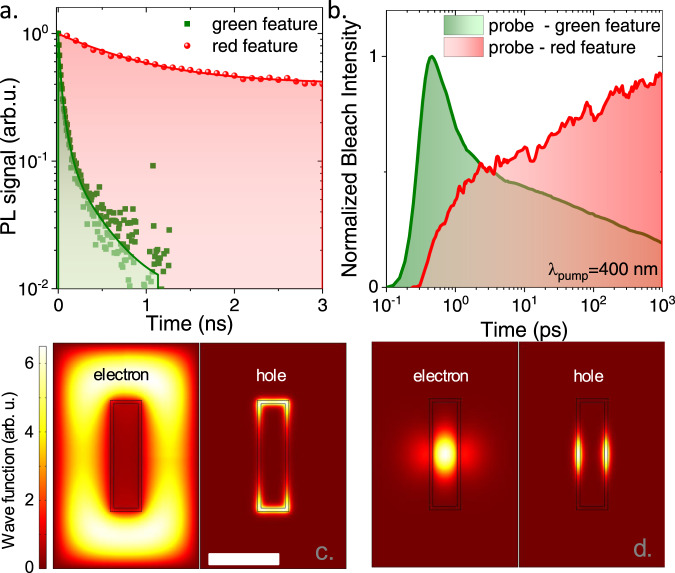
Origin of the two-color photoluminescence. **a** Time-resolved photoluminescence decays associated with the red and green features. The experimental data are scattered points, while the solid lines are exponential fits. NPL solution is excited by a 407 nm pulsed laser diode. **b** Transient absorption signal for the green and red features, while the NPLs are pumped at 400 nm. **c** Wavefunctions of the non-interacting electron and hole ground state for a NPL with large CdSe crown. **d** Same but including excitonic interactions. The color scale is the same for **c**, **d**. The shared scale bar for **c**, **d** is 20 nm.

To further reveal the nature of the two emissions, we then probe their dynamics using time-resolved PL (TRPL) and transient absorption (TA). TRPL shows (Fig. 2a) a sub-ns emission for the green emission consistent with previous reports on band-edge emission of CdSe NPLs^25^, while the red emission presents a much slower decay, resulting from the limited spatial overlap of the electron and hole wavefunctions as expected for a type-II semiconductor interface^16,18^. Low-fluence TA conducted with a 400 nm pump excitation (i.e., above CdSe band edge) shows a growth of the red bleach signal, see Fig. 2b and supplementary note 8, identified as a charge transfer band^23,26^. The transfer occurs between a typically fast (≈1 ps) and an unusually slow (hundreds of ps) timescale, which hints at the existence of a relaxation bottleneck in the formation of a charge-separated state. Ordinarily, migration of electrons and holes within nanostructures is ultrafast^23,27^. This bottleneck enables long-lived, metastable excitons in CdSe emitting at 510 nm. Its origin can be related to the absence of low-energy hole states delocalized over both CdTe and CdSe domains, which slows down phonon-mediated relaxation^15^.

We have checked using PL excitation and single particle measurement that both emissions are resulting from the same particle as discussed in supplementary note 9, we can consequently relate this bicolor emission to the electronic structure of the particle itself. To build a complete picture of the electronic structure of the CdSe/CdTe/CdSe core/crown/crown NPLs, we have performed k.p simulations. In such heterostructures, the band alignment tends to locate the ground state of a single (non-interacting) hole in the CdTe crown. The electron, in turn, is localized inside CdSe and experiences a move from within the core to the external crown when the latter is larger than 20 nm, which is indeed the experimental regime. Wavefunctions of electron and hole in the latter case are shown in Fig. 2c (see also supplementary note 10 for a systematic study). Interestingly, the inclusion of exciton (Coulomb) interactions, completely changes the charge distribution of the (indirect) ground state (Fig. 2d). The hole wavefunction migrates from the short to the long side of the CdTe crown, and the electron moves back to the core, with only moderate leakage into the external crown. This charge distribution maximizes the electron-hole overlap. The resulting exciton binding energies, which are further enhanced by the dielectric confinement, reach ≈100 meV (supplementary note 10). This is a large value for a type-II heterostructure, which anticipates non-negligible Auger relaxation rates at high incident powers. This aspect is addressed later. By contrast, the green feature corresponds to a highly excited state (over 400 meV above the indirect exciton), such that excitonic corrections are no longer relevant.

**Fig. 3:**
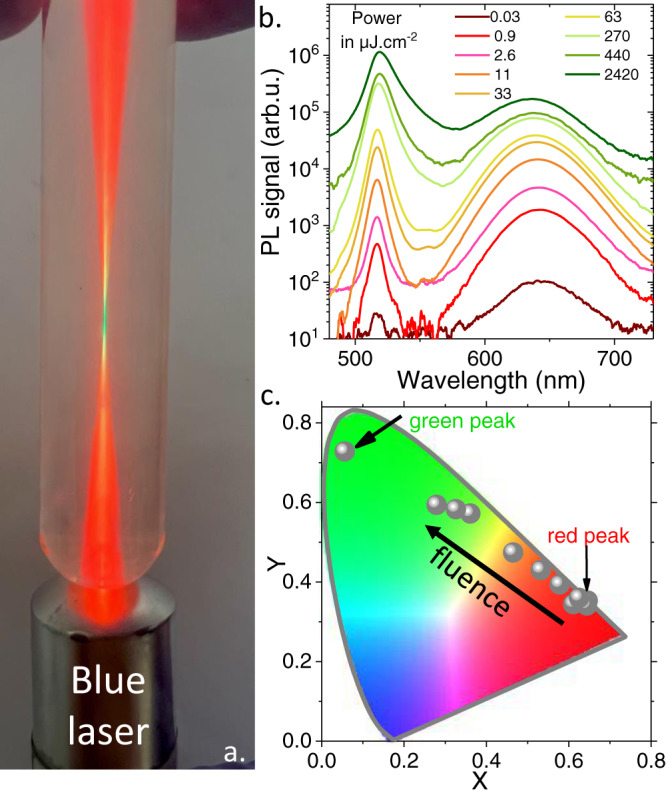
Power-dependent luminescent properties. **a** Image of a test tube containing a dilute solution of CdSe/CdTe/CdSe core/crown/crown NPLs, illuminated by a blue 405 nm laser diode. the bottom of the tube acts as a lens and luminescence at the focal point is green while being red away from it. **b** Photoluminescence spectra of CdSe/CdTe/CdSe core/crown/crown NPLs under various incident powers. **c** Chromaticity diagram and position of the PL signal resulting from the CdSe/CdTe/CdSe core/crown/crown NPLs as the incident power is increased.

A very striking feature of these NPLs is that they do not only display two contributions in their PL signal, but the relative weight of these two contributions can easily be tuned by the incident power, while the latter remains in the low irradiance regime. This power-tunable luminescence is strikingly illustrated in Fig. 3a, where a test tube containing a dilute solution of CdSe/CdTe/CdSe core/crown/crown NPLs is illuminated by a blue laser pointer. The bottom of the test tube acts as a lens and the PL signal appears red away from the focal point and green around the focal point. More quantitatively, the PL spectrum has been recorded under various incident powers in Fig. 3b. It reveals a shift from red prevailing PL at low fluences to green prevailing PL under high irradiance. Thus this power tunability can be used as a knob to shift the NPLs emission, as pointed by the displacement of the CIE coordinate as the incident power is increased, see Fig. 3c. Note that under even higher excitations (supplementary note 12), amplified spontaneous emission (ASE) can be observed^28^, but only at the green emission feature (i.e., no red stimulated emission was observed). The fluence threshold for the ASE is found to be ≈42 µJ.cm^−2^, a value above the record for core-only NPLs but which is limited in this case by the charge transfer and reabsorption losses^28–31^.

While the red emission is clearly associated with the CdSe/CdTe interface, the spatial origin of the green emission is unclear since both core and external crown have a similar emission. To break this degeneracy, we replace the external crown with an alloyed crown made of CdSSe, whose band-edge energy is larger with respect to pristine CdSe, see supplementary note 6. In this case, tricolor emission (from CdSSe, from CdSe and from CdSe/CdTe interface) is achieved and we observe that the prevailing signal results from the CdSSe external crown rather than from the core.

### Power-tunable emission

**Fig. 4:**
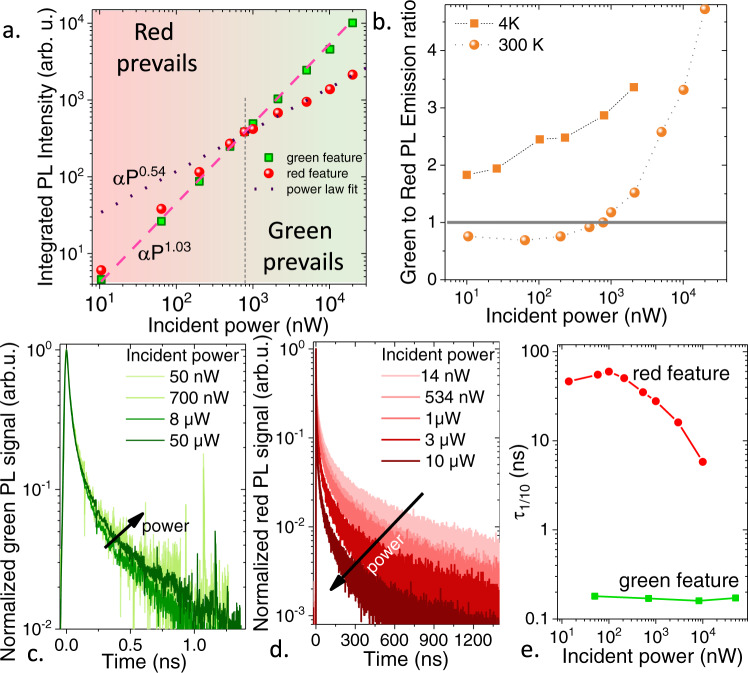
Origin of the power-dependent spectra. **a** Integrated PL intensity of the green and red features as a function of the incident power. Measurements are conducted in micro-PL configuration with the selection of emission from waist region. Power laws with exponent 1.03 and 0.54 are used to fit the green over the whole power range and the red feature for the energy range above 1 µW, respectively. **b** Ratio of the green over red integrated PL signal as a function of power at room temperature and at low temperature (*T* = 4 K). **c** Time-resolved green photoluminescence under various incident powers. **d** Time-resolved red photoluminescence under various incident powers. **e** Duration to reduce the PL signal by a factor of 10 for the green and red features as a function of the incident power.

Now coming back to the CdSe/CdTe/CdSe core/crown/crown NPLs we probe their PL spectrum as a function of incident fluence. We measure that the green PL magnitude displays a linear behavior, while the red signal saturates at high power, see Fig. 4a. Above a certain power, the scaling law of the red emission magnitude only increases with a power law close to P^0.5^. This trend is observed for all geometries of NPLs. The size of the CdTe and CdSe crown only changes the threshold power over which the green emission prevails. A larger CdTe crown favors red emission, see supplementary note 13, while a larger CdSe crown favors green emission. It is worth pointing out that at low temperature, Fig. 4b, green emission is favored. This observation agrees with a kinetic model where formed excitons diffuse from the CdSe to the interface^20^. At low temperature, this diffusive process becomes less efficient and excitons recombine radiatively in CdSe. This effect competes with faster green direct exciton emission^25^. The power-dependence of the NPL spectrum relates to the saturation of the red PL signal at high power. Interestingly, we observe that the dynamics of the green PL remain unaffected by a power increase, see Fig. 4c, while the red emission saturates as the power is increased, see Fig. 4d, e. In spite of the type-II band alignment, Auger recombination^32–34^ comes into play under high incident power and opens another non-radiative decay channel that is responsible for the saturation of the red signal with power^21,22^. For the green emission, the large volume of the external crown suppresses non-radiative Auger recombination^13,14^. Linear scaling of the green PL is preserved by radiative biexcitons (see supplementary note 13). These results also contrast with the power-tunable bicolor emission observed in quantum dots^10,35^, where saturation of one emission is the result of hole blockade. On the opposite of the small NCs, our simulation shows (see supplementary note 11) that a large core/crown NPL can accommodate at least up to 5 holes before the Coulomb blockade regime is reached. In the case of the NPLs, the power-dependence of the spectrum is more of a dynamic process, driven by the relative importance of radiative or non-radiative channels for multiexciton recombinations in the different parts of the NPL. The fact that green emission is unaffected by Auger further suggests that the Auger effect is occurring on carriers close to the band edge rather than on hot electrons.

### Bicolor electroluminescence

**Fig. 5:**
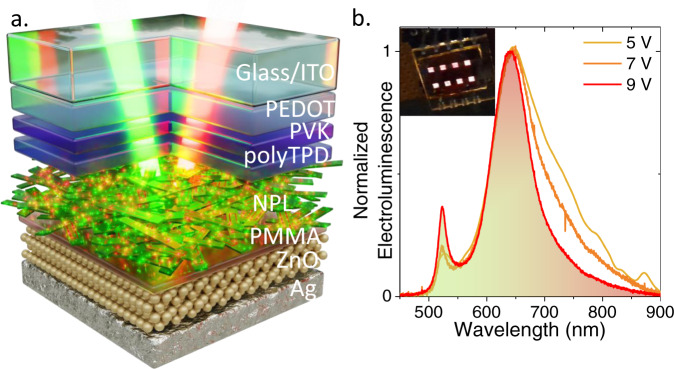
Electroluminescence from bicolor NPL. **a** Schematic of LED stack used to generate electroluminescence signals from the bicolor-emitting NPLs. **b** Electroluminescence spectra of bicolor NPL under various operating biases. The inset is a picture of 8 pixels from the LED turned on.

In the last part of the paper, we have investigated the potential of this material for multi color^12^ electroluminescence^36–40^. The diode stack is taken from Peng and coworkers^41^, since it has also proven its efficiency for the design of NPL-based LEDs^37,39^, see supplementary note 15. Briefly, the hybrid organic-inorganic LED relies on polymers (PEDOT:PSS/PVK and poly-TPD) for the hole injection, while ZnO is used for the electron injection. At the interface between the NPLs film and ZnO, we also introduce a thin PMMA layer, whose role is not to balance the charge injection, but rather to avoid PL quenching induced by the ZnO deposition. A schematic of the LED stack is shown in Fig. 5a, while an image of eight LEDs under operation is provided as an inset of Fig. 5b.

The electroluminescence spectra are given in Fig. 5b. The green and red feature appear slightly redshifted with respect to the measurements obtained in solution or using dilute film. This shift is the result of reabsorption and energy transfer occurring in dense array of particles. As for the PL, an increase of the green peak relative weight is observed as the system is brought more and more out of equilibrium. Moreover, we also notice a strong narrowing of the red peak with increased operating bias. Under large bias, the injection within or above the band edge becomes more effective, which reduces the relative weight of the emission occurring below the band gap. This result, together with the demonstration of tricolor emitting NPLs, are an important step for the design of low electrical consumption, white light-emitting LEDs for large gamut displays.

To summarize, we have designed through band alignment and scattering engineering a bicolor down-converting NPL that breaks Kasha’s rule. This CdSe/CdTe/CdSe core/crown/crown NPL combines a very narrow green emission with a deep red emission that are respectively coming from CdSe and its interface with CdTe. The relative weight of the two peaks can easily be tuned by bringing the system out of equilibrium either using a higher optical excitation power or using bias in the case of electroluminescence. The relative magnitude of the green and red emissions is controlled by the nature of the type-II interface and the volume of the external emitting crown. The power-dependence of the spectrum can be related to the saturation of the red emission at high power which itself results from non-radiative Auger recombination. This work offers an interesting alternative to nanocrystal blends for the design of a white light emitter. Future works will certainly have to focus on the stability of the material to make it compatible with operations under high flux and high temperature.

## Methods

### Chemicals

Octadecene (ODE, Alpha Aesar, 90%), cadmium acetate dihydrate (Cd(OAc)_2_.2H_2_O,Sigma-Aldrich, 98%), cadmium oxide (CdO, Strem Chemicals, 99.99%), myristic acid (Aldrich, >99%), oleic acid (OA, Alpha Aesar 90%), trioctylphosphine (TOP, Alpha Aesar, 90%), selenium (Strem Chemicals 99.99%), sulfur (Sigma-Aldrich), tellurium (Alpha Aesar, 18 + 60 mesh, 99.999%), hexane (VWR Chemicals), ethanol absolute (VWR Chemicals), methanol (VWR Chemicals)

### 1M TOP:S precursor

In a glovebox, 20 mL of TOP are mixed with 0.64 mg of S powder. The mixture is stirred for a whole night and is then stored in the glovebox for further use.

### 1M TOP:Se precursor

In a glove box, 20 mL of TOP are mixed with 1.58 mg of Se powder. The mixture is stirred for a whole night and is then stored in the glovebox for further use.

### 1M TOP:Te precursor

2.54 g of Te powder are mixed in 20 mL of TOP in a three-neck flask. The flask is kept under vacuum at room temperature for 5 min and then the temperature is raised to 100 °C. Furthermore, degassing of the flask is conducted for the next 20 min. The atmosphere is switched to nitrogen and the temperature is raised to 275 °C. The solution is stirred until a clear orange coloration is obtained. The flask is cooled down to room temperature and the color changes to yellow. Finally, this solution is transferred to a nitrogen-filled glove box for storage.

### Cd(Myr)_2_ precursor

In a 50 mL three necks flask, 2.56 g of cadmium oxide and 11 g of myristic acid are mixed and degassed at 80 °C for 30 min. The atmosphere is then switched to argon and the temperature is set at 200 °C. The mixture is heated for 40 min until the solution becomes colorless. The solution is then cooled down and 30 mL of methanol are added at 60 °C. The formed cadmium myristate is washed five times by centrifugation using methanol. The final solid is dried under vacuum at 70 °C for a whole night.

### CdSe 4ML core

In a 50 mL three necks flask, 340 mg of Cd(Myr)_2_, 24 mg of Se powder and 25 mL of ODE are added. After 20 min of degassing at room temperature, the atmosphere is switched to argon and the temperature is set at 230 °C. When the temperature reaches ~203 °C, 110 mg of Cd(OAc)_2_.2H_2_O are swiftly added. The solution is heated for 20 min and then cooled down to room temperature. At 150 °C, 500 µL of oleic acid is added. Then the obtained solution is precipitated with 20 mL of hexane and 30 mL of ethanol. The obtained pellets are washed a second time using less ethanol. The NPLs are finally redispersed in 10 mL of ODE.

### CdSe-CdTe core-crown

In a 25 mL three-neck flask, 92 mg of dried Cd(OAc)_2_, 180 µL of oleic acid and 1 mL of CdSe 4 ML core NPLs (O.D.: 1 at 512 nm for 50 µL in 3 mL of hexane) redispersed in 5 mL of ODE are degassed for 30 min at room temperature then 30 min at 80 °C. The atmosphere is switched to argon and the temperature is set at 205 °C. When the temperature is stabilized, 0.25 mL (1eq) of a solution of TOP:Te (1 M) in ODE (final concentration 0.01 M) is added at a 2 mL h^−1^ rate. After the injection, the mixture is further heated for 10 min.

### CdSe external crown

After the growth of the CdTe crown, the temperature is set at 215 °C. When the temperature is stabilized, 2 mL of TOP:Se (1 M) in ODE (final concentration 0.1 M) is added at a 1 mL h^−1^ rate. After the injection, the mixture is cooled down to room temperature and the NPLs are precipitated with hexane and ethanol for 5 min. The final pellets are redispersed in hexane.

### Transmission electron microscopy on NCs

A drop of the NC solution is drop-casted onto a copper grid covered with an amorphous carbon film. The grid is degassed overnight to reduce future contamination. A JEOL 2010F is used for the acquisition of pictures and operated at 200 kV. Complementarily, TEM/STEM observations were made on a Titan Themis 200 microscope (FEI/Thermo Fischer Scientific) equipped with a geometric aberration corrector on the probe. The microscope was also equipped with the “Super-X” systems for EDX analysis with a detection angle of 0.9 steradian. The observations were made at 200 kV with a probe current of about 35 pA and a half-angle of convergence of 17 mrad. HAADF-STEM images were acquired with a camera length of 110 mm (inner/outer collection angles were respectively 69 and 200 mrad).

### k·p modeling

Electron and heavy hole states are calculated with single-band k·p Hamiltonians. These include self-energy terms arising from the dielectric mismatch with the organic medium, as well as strain arising from the lattice mismatch between the core and the crown. The latter is obtained within the continuum elastic approximation. Excitonic interactions are obtained with a self-consistent calculation, where Coulomb integrals are obtained by integrating the Poisson equation in a dielectrically inhomogeneous system, see SI of ref. 15. for details of the model and material parameters. Many-hole calculations (Supplementary Note 11) are carried out using full configuration routines. The basis set is then formed by all possible combinations of the eight lowest spin-orbitals corresponding to the A_g_, B_1g_, B_2u_, and B_3u_ irreducible representations of the NPL point group (D_2h_) group, which provide the top-most hole states in CdTe crowns^42^. The calculations in Fig. 2 correspond to CdSe/CdTe/CdSe NPLs with 4.5 monolayer thickness. Following TEM data for a typical reference sample, the CdSe core is taken with dimensions 7 × 30 nm^2^, the CdTe crown 9 × 32 nm^2^ and the external CdSe crown 42 × 65 nm^2^. While experimental samples may have even larger external crowns, the dimensions we choose are already in the asymptotical limit for the description of the ground state (red peak), as shown in Supplementary Note 10.

### Optical spectroscopy

#### Absorption/PL measurements

UV-visible spectra are acquired with a Cary 5000 spectrometer. PL and excitation spectra are obtained with an Edinburgh instrument spectrometer. During the measurements, the NPLs are dispersed in hexane.

#### µ-photoluminescence

In order to compare the weight of each component in the PL spectra (green vs red emission) and be able to identify the processes that govern the PL power dependence, only light coming from a homogeneously excited volume should be collected (to probe equally each excitation-relaxation channel). This ‘small’ volume that roughly corresponds to the waist region of the incident beam was addressed with a confocal-like setup (afocal configuration) using the spectrometer slit (Princeton Instruments, Acton SP2750) as a spatial filter. To reach strong rejection and select the waist emission the latter was coupled to an infinity-corrected microscope objective (NA ≈ 0.6, equivalent focal length ≈6 mm) used to excite and collect light in reflection configuration. A cooled CCD (Spec10, PI) was used as a detector at the exit of the spectrometer. The excitation was supplied by a laser diode (Alphalas) operating at 407 nm (Δ*t* ≈ 70 ps). The repetition rate was adjusted at ≈300 kHz to allow complete inter-pulse relaxation of the long lifetime species responsible for the red emission. A long-pass edge filter (LP03-458RE-25, λ_cutoff_ ≈ 458 nm) from Semrock company was also placed along the detection path to suppress scattered light from the excitation beam.

#### Time-resolved PL

PL time-resolved measurements were performed using the same ‘confocal’ configuration; with two different methods. Relatively ‘long’ lifetime decays (associated with the red emission) were characterized through TCSPC using a correlator board from Picoquant (TimeHarp 260), an avalanche photodiode for the detection (MPD company, PDM module accommodating a dark count rate of ≈25 counts/s) and an Alphalas laser diode to excite the material (λ ≈ 407 nm, Δt ≈ 70 ps). The setup IRF is then measured to be ≈220 ps. Fast decays (green emission) were measured with a streak-camera (C5680 model from Hamamatsu incorporating an M5675 synchroscan unit) coupled to our Acton SP2750 spectrometer. In this configuration, the excitation (pulses of ≈2 ps duration) is the second harmonic of a Titanium-sapphire laser operating at 82 MHz and a temporal resolution of ≈ 15 ps is typically obtained depending on the dispersion of the PL through the spectrometer. Different filters combinations are used throughout time-resolved experiments: colored filters—from Corning and Thorlabs—in order to extract the spectrum part of interest as well as an additional highly selective Semrock filter (to reject photons from the laser). Due to the high emission yield of the system, a great attention was drawn during TCSPC to keep the ratio of the count rate to the excitation frequency below 2%, in order to avoid pile-up effects deleterious to the counting statistics.

All the μ-PL experiments were carried on NPLs dissolved in hexane at a relatively low concentration (O.D. ≈ 0.7 at 510 nm for a 10 mm beam path), chosen to keep a satisfying signal to noise ratio under low power excitation (nW range). It was carefully ensured that inter-NPLs effects could be discarded in the so-defined experimental conditions.

#### Transient absorption

Transient absorption measurements were performed by splitting the 800 nm fundamental of a 2 kHz 35 fs Ti:Sapphire laser (SpectraPhysics) into two branches. One branch, the pump, was frequency-doubled to 400 nm, chopped to 1 kHz, and focused on the sample. The other branch, the probe, was focused into a 2 mm sapphire disk to generate a white light supercontinuum and then focused onto the sample. The beams were overlapped spatially on the sample and the pump-probe delay was controlled by a delay stage. Spectra of the white light supercontinuum were collected under pump-on and pump-off conditions to generate ΔA data using Helios software (Ultrafast systems).

#### Amplified spontaneous emission

ASE measurements were performed using a frequency-doubled Ti:Sapphire pump excitation (400 nm, ≈35 fs, 100 Hz) focused as a 4 mm stripe on a thin-film sample of nanoplatelets. The pump power was controlled with continuous optical density wheels. Emitted light was collected normal to the pump excitation direction, focused into a fiber, and directed to a spectrometer and CCD. Films for measurements were prepared by drop-casting 9:1 hexane: octane solutions of nanoplatelets onto clean glass slides, to form smooth, reflective films. A similar configuration (using a circular lens with front-face collection) was employed to perform power-dependent PL experiments on dilute samples in cuvettes.

#### Single particle measurements

In the experiments the PL was collected with a 0.6 NA, infinity corrected objective (producing a <1 μm diameter spot) and analyzed using a 2750 Acton Spectrometer from Princeton Instruments, keeping the confocal configuration described for the TRPL. The NPLs were dispersed on ≈120 microns thick coverslips that were ‘stuck’ to the cold finger of a He-flow micro-PL cryostat (Oxford Instruments) with silver particles-based varnish to ensure a good thermal contact^42,43^. The excitation was tuned at 390 nm (SHG of a Ti-Sapphire laser delivering ps duration pulses). A dichroic filter (from Semrock company) was placed along the optical path (FF01-430/LP-25) to suppress scattering from the excitation beam; note that the scattered light from the Ti-Sapphire pump laser (THG of a diode-pumped Nd:YAG laser) could not be totally suppressed and is always present in the shown spectra as a sharp line peaking at 532 nm. Due to the poor sample stability the pump intensity was limited to a fraction of microwatt and the spectra were recorded with relatively low integration times (4–10 s typically). It is finally important noting that the strong and ≈1 s scale operating spectral diffusion was clearly identified as an important source of spectral broadening. The phenomenon was not further investigated in the course of the present project.

### Photoemission spectroscopy

#### Sample preparation for photoemission

Silicon wafers are rinsed with acetone, sonicated in acetone for 5 min. They are rinsed again with acetone and isopropanol and dried with N_2_ gun. A 5 nm layer of Cr and an 80 nm layer of Au are deposited using thermal evaporation. A diluted solution of NPLs in a mix of hexane octane (9:1) is drop-casted on the prepared substrate. After drying, the film is dipped in a solution of EDT (1% in acetonitrile) for 1 min. The procedure is repeated 3 times. The film final is stored under inert atmosphere before its introduction into the preparation chamber, where it is degassed for at least two hours and then transferred to the analysis chamber.

#### Photoemission data acquisition

XPS experiments are carried out on the TEMPO beamline from SOLEIL French synchrotron facility. The photon sources were HU80 and HU44 Apple II undulators set to deliver linearly polarized light. The photon energy is selected using a high-resolution plane grating monochromator. During the XPS measurements, the photoelectrons are detected at 0° from the sample surface normal $\vec{{{{{{\bf{n}}}}}}}$ and at 44° from the polarization vector $\vec{{{{{{\bf{E}}}}}}}$. The spot size is 100 × 80 μm². The signal is acquired onto a MBS A-1 photoelectron analyzer equipped with a delay line detector developed by Elettra^44^.

#### Energy calibration

Valence and secondary electron cut-off measurements are conducted at 150 eV which corresponds to a surface sensitive condition, while core levels are typically acquired using a 700 eV photon energy. The photon energy is precisely measured using the first and second order of a given core level using the formula: $h{\nu }_{{{\exp}}}={{{{{{\rm{KE}}}}}}}_{{2}^{{{{{{\rm{nd}}}}}}}}-{{{{{{\rm{KE}}}}}}}_{{1}^{{{{{{\rm{st}}}}}}}}$, where KE stands for kinetic energy. The work function of the analyzer (${{{{{{\rm{WF}}}}}}}_{A}$) is determined by measuring the kinetic energy of electrons at the Fermi level from a gold reference sample. The binding energy of the Fermi level is set to 0 eV. ${{{{{{\rm{WF}}}}}}}_{A}=h{\nu }_{{{\exp}}}-{{{{{{\rm{KE}}}}}}}_{{{{{{\rm{Fermi}}}}}}}$

#### Valence band measurement

We determine the value of ${V}_{B}-{E}_{F}$by looking at high KE electrons. We measure the highest kinetic energy available (${{{{{{\rm{KE}}}}}}}_{{{{{{\rm{VB}}}}}}}$) and extract ${V}_{B}-{E}_{F}$ with the formula: ${V}_{B}-{E}_{F}=h{\nu }_{{{\exp }}}-{{{{{{\rm{KE}}}}}}}_{{{{{{\rm{VB}}}}}}}-{{{{{{\rm{WF}}}}}}}_{A}$.

#### Core level

All spectra are calibrated in energy by shifting them so that the Fermi level of metallic samples presents null binding energy. A Shirley background is subtracted in all core level spectra. The core level is then fitted using a Voigt curve, which displays a typical 0.8 eV full width at half maximum.

#### Work function measurement

In order to measure the work function, which is the difference in energy between vacuum level and Fermi level, we look for the cut-off of secondary electrons (${{{{{{\rm{KE}}}}}}}_{{{{{{\rm{Cut\; off}}}}}}}$). We start by polarizing the sample using an 18 V (${{{{{{\rm{Pol}}}}}}}_{{{{{{\rm{Bias}}}}}}}$) voltage supply (TDK lambda) and we look for the energy edge of the lowest kinetic energy photoelectrons. The work function is deduced with the formula: ${{{{{{\rm{WF}}}}}}}_{{{{{{\rm{Sample}}}}}}}={{{{{{\rm{KE}}}}}}}_{{{{{{\rm{Cut\; off}}}}}}}-{{{{{{\rm{Pol}}}}}}}_{{{{{{\rm{Bias}}}}}}}$.

### LED fabrication

#### Materials for LED

PEDOT:PSS (poly(3,4- ethylenedioxythiophene) polystyrene sulfonate, Al 4083, M121, Ossila), Poly-TPD (Poly(N,N’-bis-4- butylphenyl-N,N’-bisphenyl)benzidine, Ossila), PVK (Poly(9-vinyl) carbazole, average Mn 25,000–50,000, Aldrich), PMMA (polymethyl metacrylate, Arkema), chlorobenzene (anhydrous, 99.8%, Sigma-Aldrich), m-xylene (anhydrous, ≥99%, Sigma-Aldrich), epoxy-glue (Ossila), zinc acetate dihydrate (<97%, Alfa Aesar), tetramethylammonium hydroxide pentahydrate (TMAOH, 98%, Alfa Aesar), dimethyl sulfoxide (DMSO, ≥99.9%, Sigma-Aldrich), ethyl acetate (VMR), ethanol absolute anhydrous (VMR), octane (VWR, technical) and acetone (VWR). All the materials were used as received.

#### Synthesis of ZnO nanoparticles

The procedure is taken from ref. 41. In flask A, 3 mmol of zinc acetate are dissolved in 30 mL of DMSO by vigorous stirring. At the same time, 5.5 mmol of TMAOH are dissolved with 10 mL of ethanol in flask B. Then the contents of the two flasks are mixed and stirred for 24 h under ambient conditions. The reaction mixture turns whitish during the first few seconds and becomes clear soon after. ZnO particles are precipitated by ethyl acetate and redisperse in ethanol. 160 µL of 2-ethanolamine are added to stabilize the nanoparticles before they are precipitated and redispersed with ethyl acetate and ethanol respectively again. Finally, the ZnO nanoparticles in ethanol are filtered using a 0.22 µm PTFE filter.

#### ITO substrate patterning

ITO substrates (30 Ω/sq) are cut into 15 mm × 15 mm pieces and cleaned by sonication in acetone for 5 min. After sonication, the substrates are rinsed with acetone and isopropanol before being dried completely with N_2_ flow. The substrates are further cleaned with O_2_ plasma for 5 min to remove organic residuals on the surface. After cleaning, TI-Prime and AZ 5214E photoresist are sequentially spin-coated on the surface of ITO substrates at the rate of 4000 rpm for 30 s and baked at 110 °C for 120 s and 90 s, respectively. In the next stage, a mask aligner is used to expose the substrates to UV light for 20 s through a lithography mask (1 mm width). A photoresist is then developed using AZ 726 developer for 20 s before rinsing with deionized water and drying with N_2_ flux. After another 5-minute plasma cleaning, the substrates are etched in a 25% HCl (in water) bath for 10 min at 40 °C before they are dipped immediately in deionized water. Finally, the lift-off is conducted in an acetone bath. Before being used, the patterned ITO substrates are cleaned with acetone and isopropanol first and put under plasma for 10 min.

#### LED Fabrication

PEDOT:PSS solution (filtered through 0.45 µm filter) is spin-coated on a patterned ITO glass electrode at 4000 rpm for 60 s and annealed at 140 °C for 10 min in air. Inside a nitrogen-filled glovebox, Poly-TPD (8 mg mL^−1^ in chlorobenzene), PVK (1.5 mg mL^−1^ in m-xylene), NPLs (in a mix of hexane/octane (9:1)), PMMA (5 mg mL^−1^ in acetone) and ZnO nanoparticles are successively spin-coated at 2000 rpm for 45 s on the PEDOT: PSS-coated substrate. After the deposition of Poly-TPD, the sample is annealed at 110 °C for 20 min, and for PVK the annealing is at 170 °C for 30 min. Finally, 80 nm of Ag is deposited on top of the ZnO using a shadow mask by thermal evaporation. The thickness of NPL and ZnO layers are 18 nm and 80 nm respectively, as obtained by profilometry. The devices are encapsulated inside the glove box with a piece of glass by epoxy-glue. The size of the pixel is 1 mm^2^ which is the overlap of ITO and Ag electrodes.

#### LED characterization

The EQE of the device is determined according to the method from ref. 45. Considering the Lambertian emission of LED device, the flux leaving the device directly can be described as ${F}_{{{{{{\rm{ext}}}}}}}={\int }_{0}^{\pi /2}2\pi {L}_{0}{{\cos}}\theta {{\sin}}\theta d\theta=\pi {L}_{0}$, with ${L}_{0}$ the flux per solid angle of light leaving the device in the forward direction. Since the solid angle from the photodetector to the light source is $\Omega=\frac{{S}_{1}}{{l}^{2}}$ with ${S}_{1}$ the area of the detector and $l$ the distance between the light source and detector, then ${L}_{0}=\frac{{P}_{{\rm {det }}}}{\Omega }=\frac{{P}_{{{{{{\rm{d}}}}}}{{{{{\rm{et}}}}}}}{l}^{2}}{{s}_{1}}$ and ${F}_{{{{{{\rm{ext}}}}}}}=\frac{{\pi P}_{{{{{{\rm{d}}}}}}{{{{{\rm{et}}}}}}}{l}^{2}}{{s}_{1}}$ . The number of photons emitted per second to the forward direction can then be calculated by ${N}_{P}=\frac{{F}_{{{{{{\rm{ext}}}}}}}}{h\nu }=\frac{{\pi P}_{{{{{{\rm{d}}}}}}{{{{{\rm{et}}}}}}}{l}^{2}\lambda }{{s}_{1}{hc}}$, with $\lambda$ the wavelength of electroluminescence, $h$ the Plank’s constant and $c$ the speed of light. The number of electrons injected per second can be obtained by ${N}_{P}=\frac{I}{e}$, with $I$ the current flow of the device. Thus, the EQE can be calculated as ${{{{{\rm{E}}}}}}{{{{{\rm{QE}}}}}}=\frac{{N}_{p}}{{N}_{e}}=\frac{{\pi P}_{d{et}}{l}^{2}\lambda e}{{s}_{1}{hcI}}$ . The irradiance of the device is $R=\frac{{F}_{{{{{{\rm{ext}}}}}}}}{{s}_{2}}=\frac{{\pi P}_{{{{{{\rm{d}}}}}}{{{{{\rm{et}}}}}}}{l}^{2}}{{s}_{1}{s}_{2}}$, with ${S}_{2}$ the area of the pixel. The luminance *L* of the device is $L=\frac{683.V\left(\lambda \right).{F}_{{{{{{\rm{ext}}}}}}}}{{\pi .S}_{2}}$, with $V\left(\lambda \right)$ the function of photonic eye sensitivity. For the characterization, we collected current-voltage-luminance characteristics with a Keithley K2634B sourcemeter unit and a PM100A powermeter coupled with the S120C Si detector from Thorlabs. Knowing that the working diameter of the detector area is 9.5 mm and assuming the distance between detector and device to be 6.5 mm, the geometry-related value is $\frac{{l}^{2}}{{s}_{1}}\approx\, 0.6$.

### Reporting summary

Further information on research design is available in the Nature Research Reporting Summary linked to this article.

## Supplementary information

Supplementary Information

Reporting Summary

## Data Availability

The data that support the findings of this study are available from the corresponding author upon request.

## Code availability

The custom code used to conduct the electronic structure modeling is available from the corresponding author upon request.
