# Supplementary material for: Double-crowned 2D semiconductor nanoplatelets with bicolor power-tunable emission
Source: Nat Commun. 2022 Aug 30;13:5094. doi: 10.1038/s41467-022-32713-2 (PMC9427944; doi:10.1038/s41467-022-32713-2)
Supplement: Supplementary file 1 — Supplementary Information [file 41467_2022_32713_MOESM1_ESM.docx]

*Supplementary information*

**Double-crowned 2D semiconductor nanoplatelets with bicolor power-tunable emission**

Corentin Dabard^1^, Victor Guilloux^2^, Charlie Gréboval^2^, Hong Po^1^, Lina Makke^1^, Ningyuan Fu^1^, Xiang Zhen Xu^1^, Mathieu G. Silly^3^, Gilles Patriarche^4^**,** Emmanuel Lhuillier^2^, Thierry Barisien^2^, Juan I. Climente^5^, Benjamin T. Diroll^6^, Sandrine Ithurria^1*^

^1^ Laboratoire de Physique et d’Etude des Matériaux, ESPCI-Paris, PSL Research University, Sorbonne Université Univ Paris 06, CNRS UMR 8213, 10 rue Vauquelin 75005 Paris, France.

^2^ Sorbonne Université, CNRS, Institut des NanoSciences de Paris, INSP, F-75005 Paris, France.

^3^ Synchrotron SOLEIL, L'Orme des Merisiers, Départementale 128, 91190 Saint-Aubin, France

^4^ Centre de Nanosciences et de Nanotechnologies, CNRS, Université Paris-Saclay, C2N, Palaiseau 2110, France.

^5^ Departament de Quimica Fisica i Analitica, Universitat Jaume I, E-12080, Castello de la Plana, Spain

^6^ Center for Nanoscale Materials, Argonne National Laboratory, Lemont, Illinois 60439, United States.

*To whom correspondence should be sent: [sandrine.ithurria@espci.fr](mailto:sandrine.ithurria@espci.fr)

Table of content

[Supplementary note 1: CdSe nanoplatelets growth 2](#_Toc110434316)

[Supplementary note 2: Attempt for bicolor NPL 2](#_Toc110434317)

[Supplementary note 3: Effect of CdTe crown size 5](#_Toc110434318)

[Supplementary note 4: Effect of CdSe crown size 6](#_Toc110434319)

[Supplementary note 5: Microscopic characterization of the bicolor emitting NPL 8](#_Toc110434320)

[Supplementary note 6: Tricolor emitting nanoplatelet 10](#_Toc110434321)

[Supplementary note 7: Electronic structure analysis of the core/crown/crown NPL revealed by photoemission 12](#_Toc110434322)

[Supplementary note 8: Evidence for coupling of the green and indirect exciton 14](#_Toc110434323)

[Supplementary note 9: Origin of the two luminescence 16](#_Toc110434324)

[Supplementary note 10: Electronic structure of the electron and hole ground state 19](#_Toc110434325)

[Supplementary note 11: Absence of hole blockade in the CdTe crown 22](#_Toc110434326)

[Supplementary note 12: Stimulated emission and multiexciton emission 25](#_Toc110434327)

[Supplementary note 13: Effect of geometrical factor on the power threshold relative to green emission prevalence 27](#_Toc110434328)

[Supplementary note 14: LED characterization 28](#_Toc110434329)

[Supplementary references 29](#_Toc110434330)

# Supplementary note 1: CdSe nanoplatelets growth

Because of the high sensitivity of the eye in the green region, narrow green emission is a critical parameter to design a large color gamut display.

The 4.5 ML CdSe NPL presents a narrow PL signal at 510 nm with a full width at half maximum of 12 nm. This narrow green PL results from their specific growth mechanism that prevents roughness along its only confined direction. As a result, there is no inhomogeneous broadening.

In the following part, these 4.5 ML CdSe NPLs will be used as a central building block of a planar heterostructure, where the surrounding of the CdSe core is used to generate red emission.

**Supplementary figure 1a** shows a TEM image of the NPLs, they present lateral dimensions of 7nm x 33 nm (**Supplementary figure 1c**). Their thickness corresponds to 4.5 ML of CdSe (*ie* 4 Se planes surrounded by 5 Cd planes). These NPLs display the first excitonic features at 510 nm,^1^ see **Supplementary figure 1b**.

**
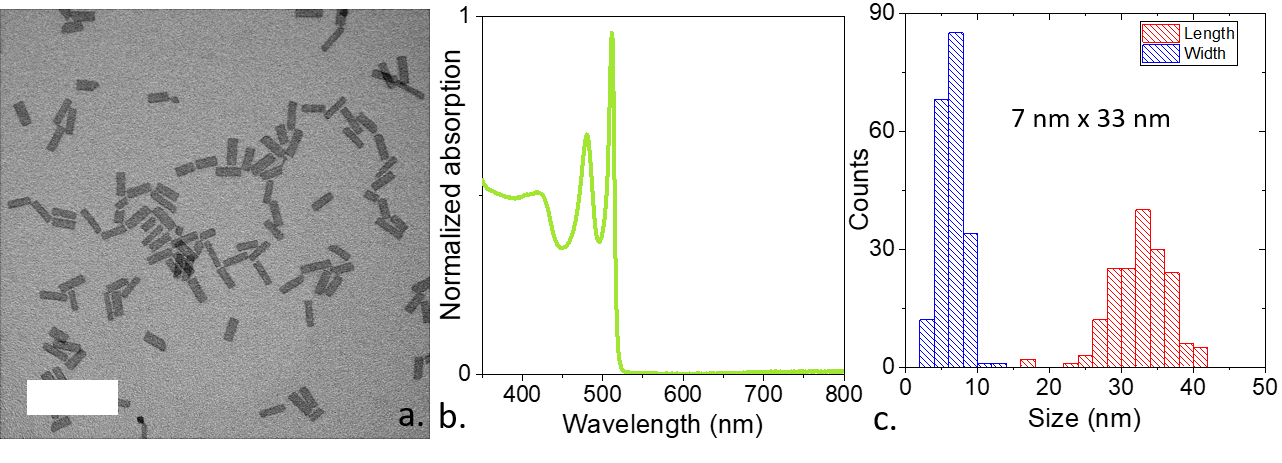
**

# *Supplementary figure 1 Synthesis of 4.5 MLs CdSe cores. a. TEM picture of the CdSe 4.5 ML cores. The scale bar is 100 nm. b. Absorption spectrum and c. Size repartition of the CdSe 4.5 ML cores.*

# Supplementary note 2: Attempt for bicolor NPL

- **Bicolor emission from CdSe/CdS/CdSeTe core/crown/crown NPL**

A possible strategy to design a bicolor emitting NPL is to combine two emitting materials separated by an optically active barrier, see **Supplementary figure 2**a. CdS, with its wide band gap can easily acts as a barrier that uncouples two narrower band gap materials, see the schematic band diagram in **Supplementary figure 2**b. CdS nevertheless still absorbs blue photons and acts as a light concentrator. The green emission is obtained from CdSe, while the red is obtained from a narrower band gap CdSeTe alloy. The exact content of the alloys can be used to further tune the energy of the red emission. For Te content above 40 % we obtain a bicolor emission, see **Supplementary figure 2**c-f. But, the red signal drastically prevails and is not deep red enough to be used as a red down converter.


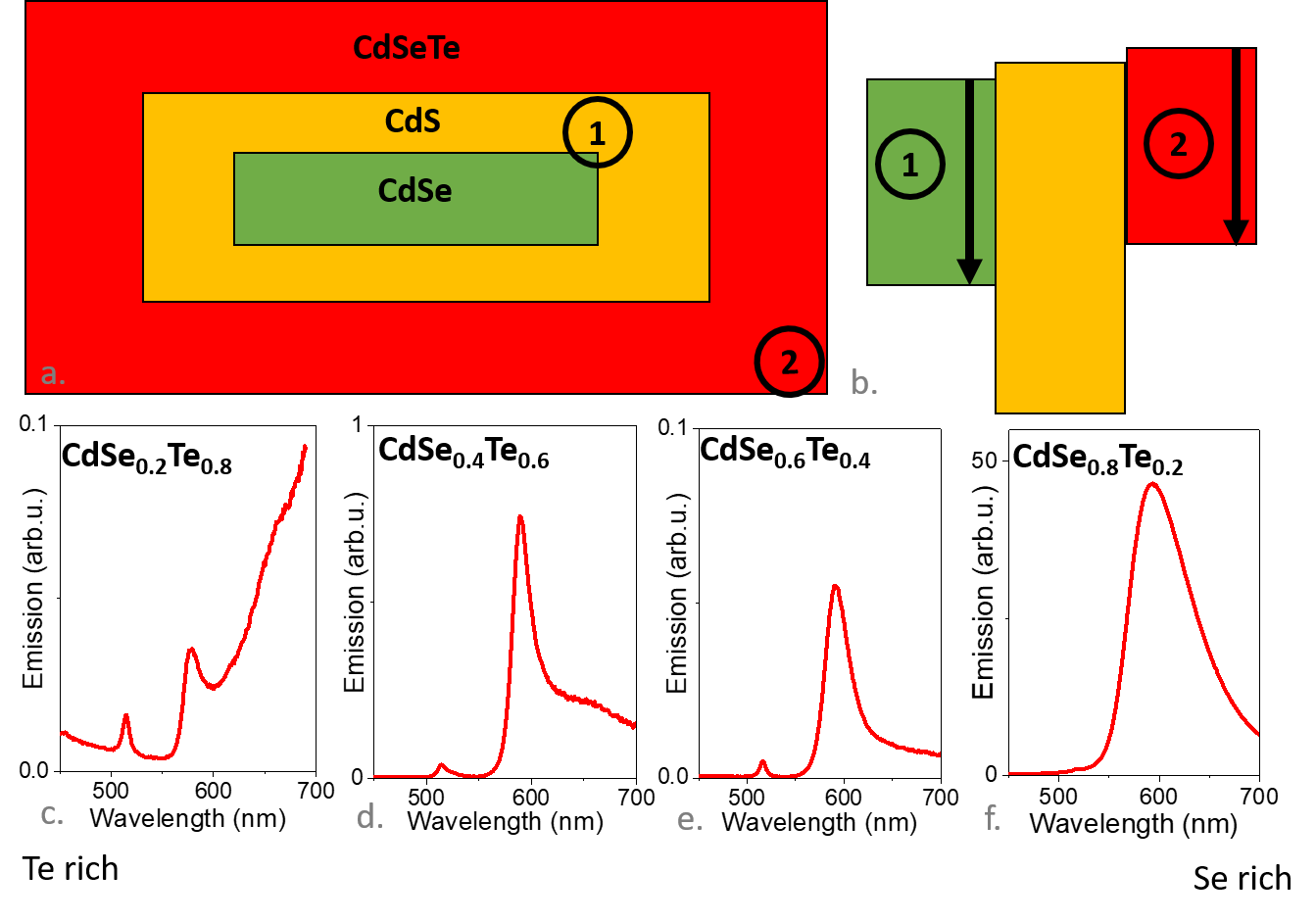


**Supplementary figure 2 CdSe/CdS/CdSeTe core/crown/crown NPL.** a. Schematic of the 4.5 ML CdSe/CdS/CdSeTe core/crown/crown NPL. b. Band alignment of the CdSe/CdS/CdSeTe core/crown/crown NPL. c-f. Photoluminescence spectra for CdSe/CdS/CdSeTe core/crown/crown NPL with various Te contents in the final crown.

- **Bi color emission from CdSe/CdS/CdSe/CdTe core/crown/crown/crown NPL**

A second strategy to generate a red signal can be based on interfacial emission resulting from a type II band alignment such as the one obtained in a CdSe/CdTe interface, see **Supplementary figure 3**b. Compared to the case depicted in **Supplementary figure 2**, the central part is preserved but the external alloy is replaced by a CdSe/CdTe crown/crown, see **Supplementary figure 3**a and c-d for TEM images. In this case, the red PL emission appears around 650 nm corresponding to a deep red color, see **Supplementary figure 3**e. However, in all cases, the red photoluminescence (PL) prevails compared to the green one. The CdSe/CdTe interface is placed on the outer part of the NPL and this generates a large perimeter for the interfacial emission. This is why in the main text, the targeted structure includes the CdTe interface closer to the center of the NPL


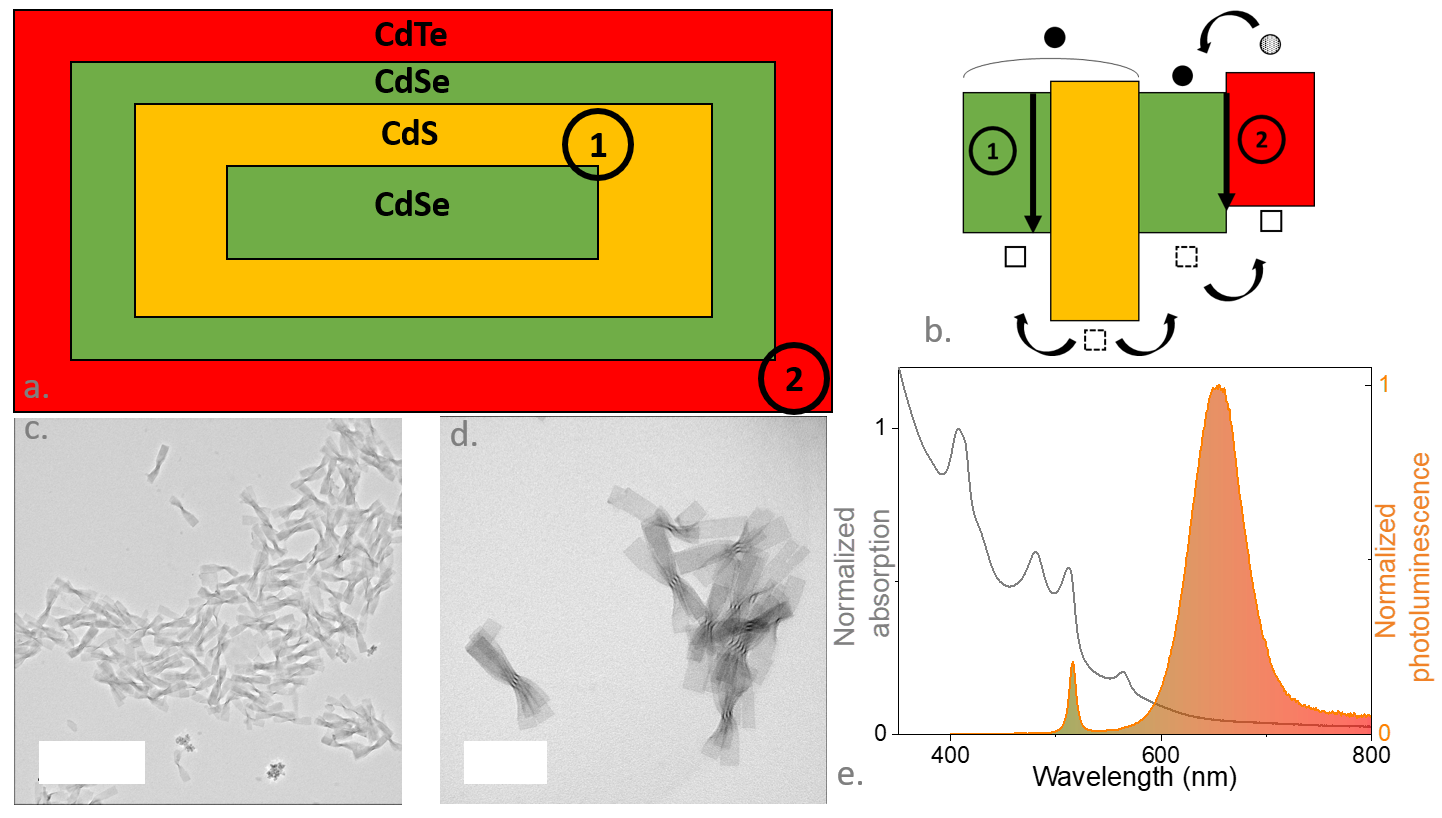


**Supplementary figure 3 CdSe/CdS/CdSe/CdTe core/crown/crown/crown NPLs.** a. Schematic of the CdSe/CdS/CdSe/CdTe core/crown/crown/crown NPL. b. Band alignment of the CdSe/CdS/CdSe/CdTe core/crown/crown/crown NPL. c and d are TEM images of the CdSe/CdS/CdSe/CdTe core/crown/crown/crown NPL. scale bars are respectively 400 nm and 100 nm large. e. Absorption and photoluminescence spectra of the 4.5 ML CdSe/CdS/CdSe/CdTe core/crown/crown/crown NPL.

# Supplementary note 3: Effect of CdTe crown size

**Supplementary figure 4** discusses the effect of a CdTe crown around the CdSe core introduced in **Supplementary figure 1**. As CdTe crown is grown, we observe a new feature in the absorption spectrum that appears around 550 nm, see **Supplementary figure 4a**. In the core crown geometry, the absorptions of the two materials sum-up and the obtained absorption spectrum combines the one of the CdSe core with the one of CdTe with the same thickness. On the other hand, the emission of such NPL is driven by the type II interface and thus appears redshifted with respect to the absorption of both materials, see **Supplementary figure 4b** and inset of **Supplementary figure 4c.** We notice that PL redshifts, when more CdTe is introduced. Actually, TEM images (**Supplementary figure 4c)** reveal that the introduced amount of CdTe only results in the growth of a few nm crown of CdTe. Consequently, there is lateral quantum confinement occurring in the CdTe crown that is responsible for the redshift observed as the CdTe amount is increased.

**
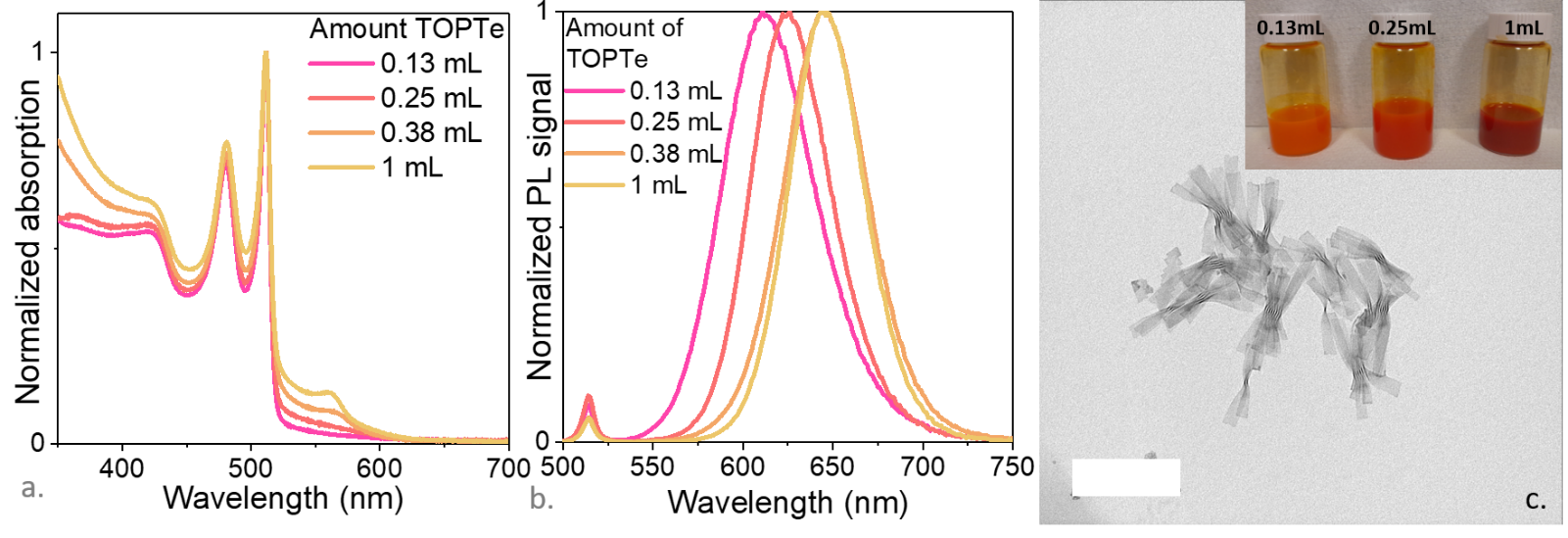
**

**Supplementary figure 4 Synthesis of the first crown made of CdTe and its influence on the red emission**. a. Absorption spectra of the NPLs after the growth of the first crown made of CdTe, according to the quantity of TOPTe (0.01 M in ODE) injected. For sake of clarity, the absorption spectra have been normalized to the first excitonic peak. b. The corresponding emission spectra according to the quantity of TOPTe (0.01 M in ODE) injected. For sake of clarity, the emission spectra have been normalized to the red emission. c. TEM picture of the core/crown/crown NPLs obtained with 0.25 mL of TOPTe (0.01 M in ODE). The scale bar is 200 nm. The inset shows the solution obtained after the growth of the second crown with various amounts of TOPTe (from left to right: 0.13 mL, 0.25 mL and 1 mL).

# Supplementary note 4: Effect of CdSe crown size

The last step of the growth consists in the growth of a CdSe crown and leads to the formation of a NPL with a structure spatially depicted in **Supplementary figure 5**a and with a band alignment as shown in **Supplementary figure 5**b. The latter, in particular, presents a type II band alignment which is used to generate the red emission.


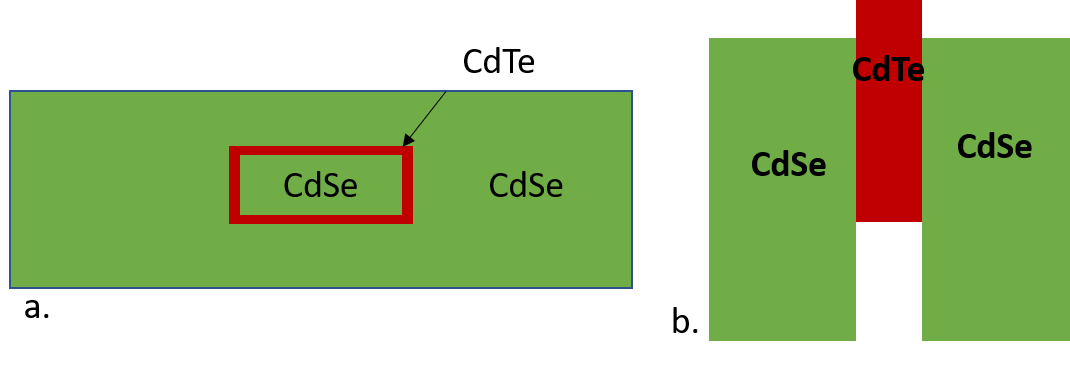


**Supplementary figure 5 CdSe/CdTe/CdSe core/crown/crown NPL.** a. Schematic of the 4.5 ML CdSe/CdS/CdSeTe core/crown/crown NPL. b. Band alignment of the CdSe/CdS/CdSeTe core/crown/crown NPL.

When introducing more precursors for the CdSe crown, we observe from TEM an increase in the lateral size of the NPLs, see **Supplementary figure 6**d-f. When the external CdSe crown is small, we mostly notice the red emission from the CdSe/CdTe interface, see **Supplementary figure 6**a. When the CdSe crown is further grown, the relative weight of the green emission increases, see **Supplementary figure 6**b and c. On the other hand, the two emission peaks remain at the same energy.

**
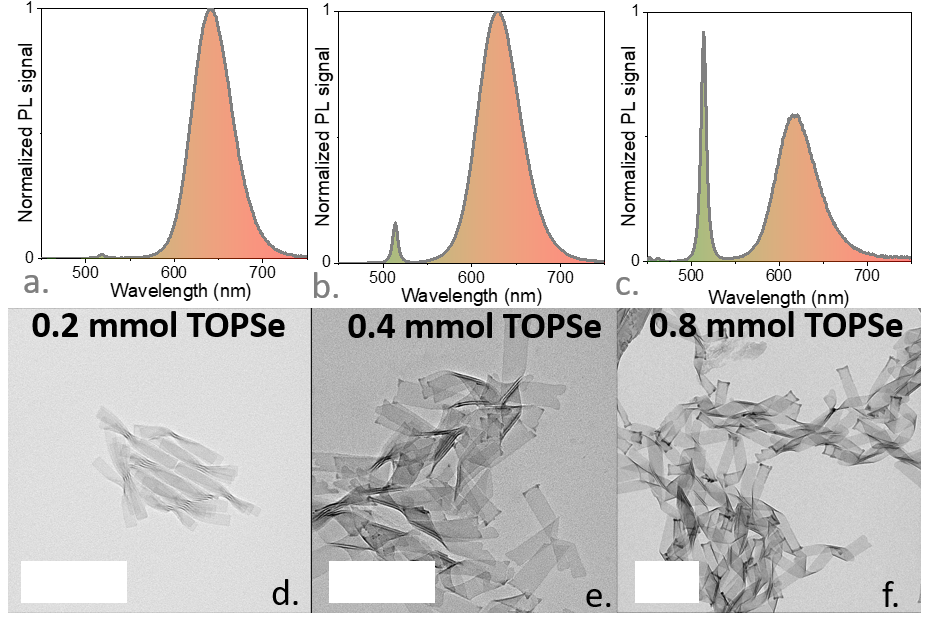
**

**Supplementary figure 6 Synthesis of the second crown made of CdSe and its influence on the ratio between the green and red emissions.** a. (resp. b. and c.) Emission spectrum of the core/crown/crown NPLs with the first crown of CdTe made with 0.25 mL of TOPTe (0.01 M in ODE) and the second crown of CdSe made with 2 mL (resp. 4 mL and 8 mL) of TOPSe (0.1 M in ODE. d. (resp. e. and f.) corresponding TEM pictures with the emission spectrum presented in a. (resp. b. and c.). All the scale bars are 200 nm.

# Supplementary note 5: Microscopic characterization of the bicolor emitting NPL

In **Supplementary figure 7**, we have performed EDX mapping to reveal the localization of Cd, Se and Te. While Se and Cd show a clear correlation with the contrast image, the Te signal is too weak to unveil the exact location of the first crown.


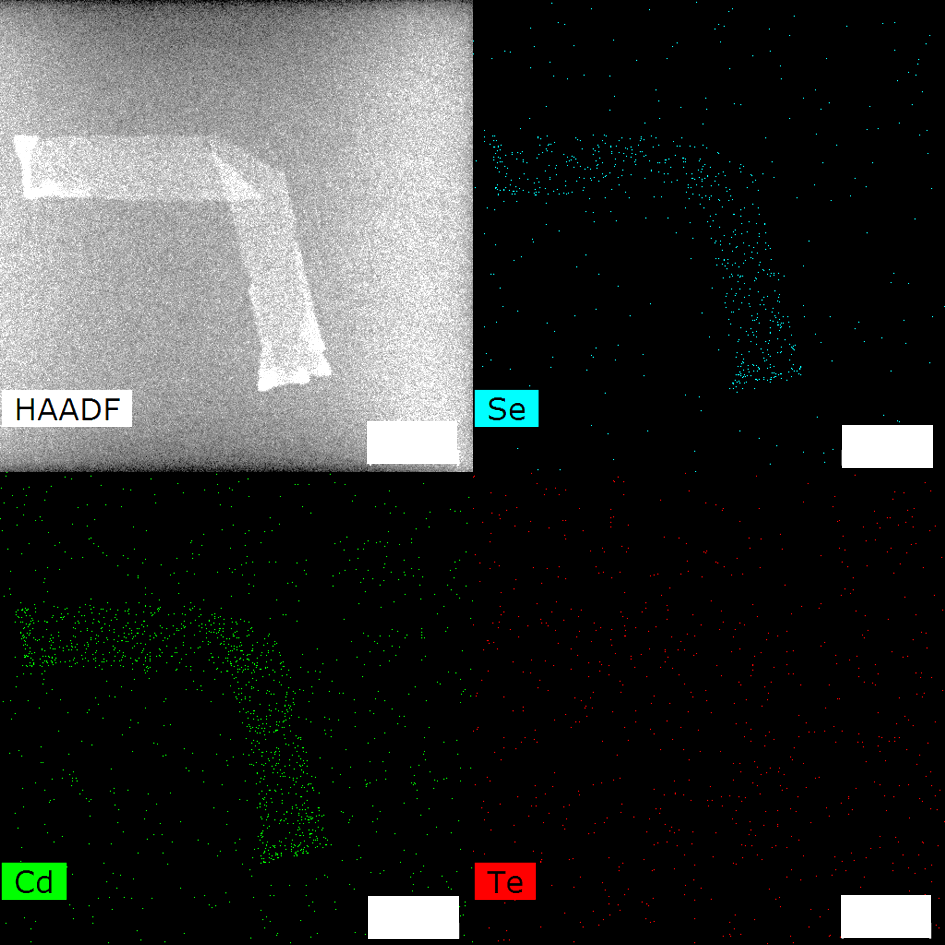


**Supplementary figure 7 EDX mapping of a single particle**. Top left is STEM HAADF image of a CdSe/CdTe/CdSe core/crown/crown NPL. top right, bottom left and bottom righr respectively show the EDX mapping of the same area. Scale bar is 50 nm.

To nevertheless reveal the localization of the CdSe core and first CdTe crown, we can heat the NPLs in presence of 1 mL of oleylamine for 10 min at 150°C. The CdTe tends to be dissolved by this procedure, unveiling the actual location of the first crown, see **Supplementary figure 8**. It appears that similarly to what has been observed for dot in rods structure^2^ the core can be shifted from the particle center.


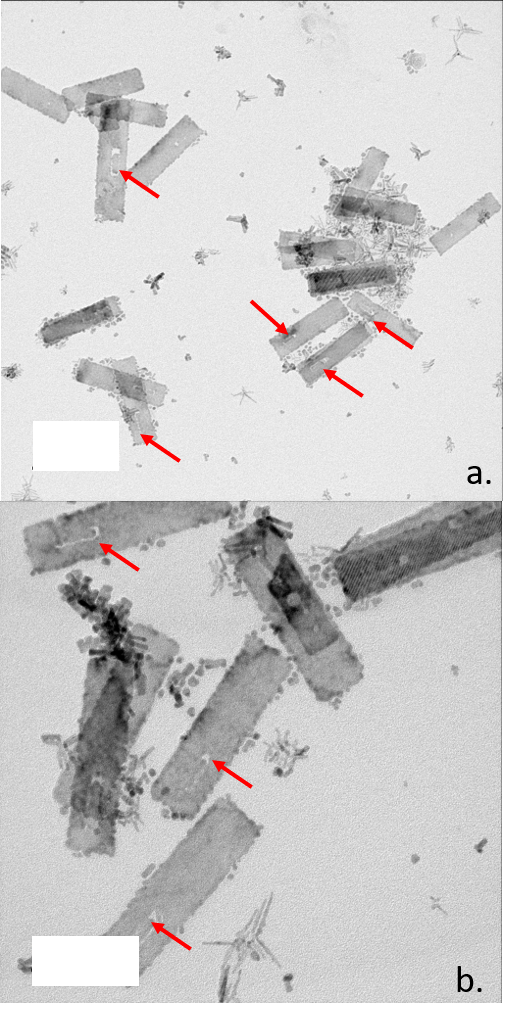


**Supplementary figure 8 TEM image of the CdSe/CdTe/CdSe core/crown/crown NPL after being heated in oleylamine**. In part a scale bar is 200 nm, while in part b it is 100 nm. Red arrows highlight where CdTe has been etched thus unveiling localization of the CdTe first crown.

# Supplementary note 6: Tricolor emitting nanoplatelet

To reveal the spatial origin of the green emission (core vs external crown) we have designed a structure for which the emission of the two areas is non-degenerated. The core is still made of CdSe and of a first crown of CdTe. We then grow a thin crown of CdSe, by doing so, we preserve the band alignment and dielectric environment of the CdTe crown. Then, we add a large external crown of CdSSe which presents a slightly higher band gap (500 nm) compared to the 510 nm gap associated with the CdSe NPL. A schematic of the NPL is given in **Supplementary figure 9**a and the associated TEM is shown in **Supplementary figure 9**b. PL spectrum now reveals 3 contributions. One is around 500 nm coming from the CdSSe external crown, one from CdSe at 510 nm and the interfacial emission around 650 nm. As excitation power is increased (see **Supplementary figure 9**d and e), this is the bluest peak resulting from the external crown which relative magnitude rises.


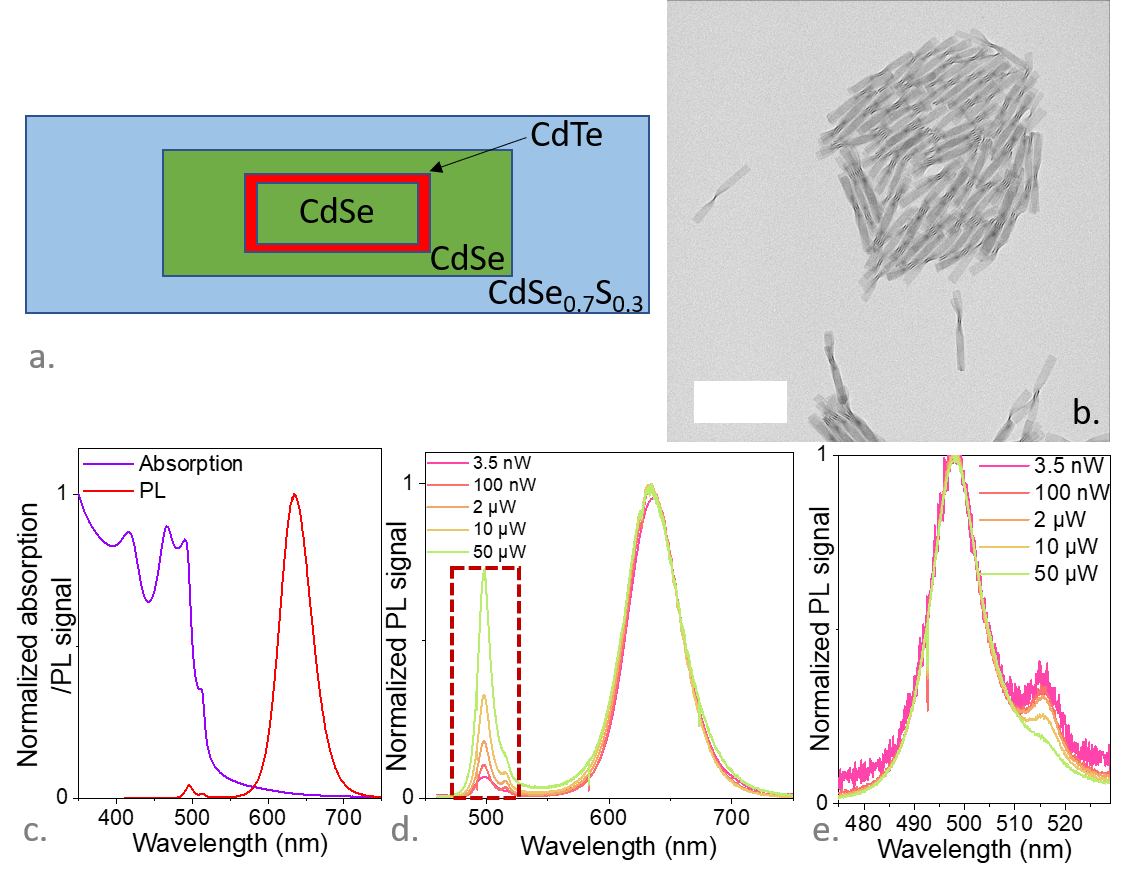


**Supplementary figure 9 Synthesis of a core/multi-crown NPL with a tricolor emission**. a. Scheme of the core/multi-crown synthetized. b. Corresponding TEM pictures of the NPLs obtained after the injection of 0.25 mL of TOPTe (0.01 M in ODE) for the first crown, 0.5 mL of TOPSe (0.1M in ODE) and 1.5 mL of a mix TOPSe/S (70/30) (0.1 M in ODE). The scale bar is 200 nm. c. Absorption (in purple) and emission (in red) spectra of the corresponding core/multi-crown NPLs. For more clarity, the absorption spectrum has been normalized at 350nm and the emission spectrum at the maximum emission. d. Emission spectra of the core/multi-crown NPLs according to the pump fluence. For more clarity, the emission spectra have been normalized to the red emission. e. Emission spectra of the core/multi-crown NPLs according to the pump fluence centered on the two emissions around 500nm. For more clarity, the emission spectra have been normalized to the bluer emission.

# Supplementary note 7: Electronic structure analysis of the core/crown/crown NPL revealed by photoemission

X-ray photoemission (XPS) has been used to probe the chemical environment of the NPLs. Due to the relatively wide band gap of NPLs, the initial long insulating native ligands are exchanged withshort thiols (ethanedithiol) this procedure enables to measure XPS while no charging effect was observed. It also explains the reason why S contribution is observed on the overview spectrum of Figure 1 in the main text.

With XPS the presence of Te is clearly confirmed, see **Supplementary figure 10**a. The Te 3d 5/2 state (Spin-orbit coupling 10.4 eV and full width at half maximum 1.2 eV) presents two contributions appearing at 572.8 eV and 574.2 eV.

The Cd 3d 5/2 state (Spin orbit coupling 6.7 eV and full width at half maximum 0.8 eV) presents also two contributions appearing at 405.1 eV and 405.7 eV, see **Supplementary figure 10**b. the main contributions can be associated with Cd coupled to Se atoms

Cd coupled with Te has probably a very low contribution and cannot explain the second contribution since Te is less electronegative than Se, the second contribution should appear at lower binding energy. Thus, we can associate the second peak to surface Cd coupled with the thiol used to make the film conductive and avoid charging. S being more electronegative than Se, this contribution appears for increased binding energy

The Se 3d 5/2 state (Spin-orbit coupling 1.1 eV and full width at half maximum 0.8 eV) presents also two contributions appearing at 53.8 eV and 55.7 eV, see **Supplementary figure 10**c.

**
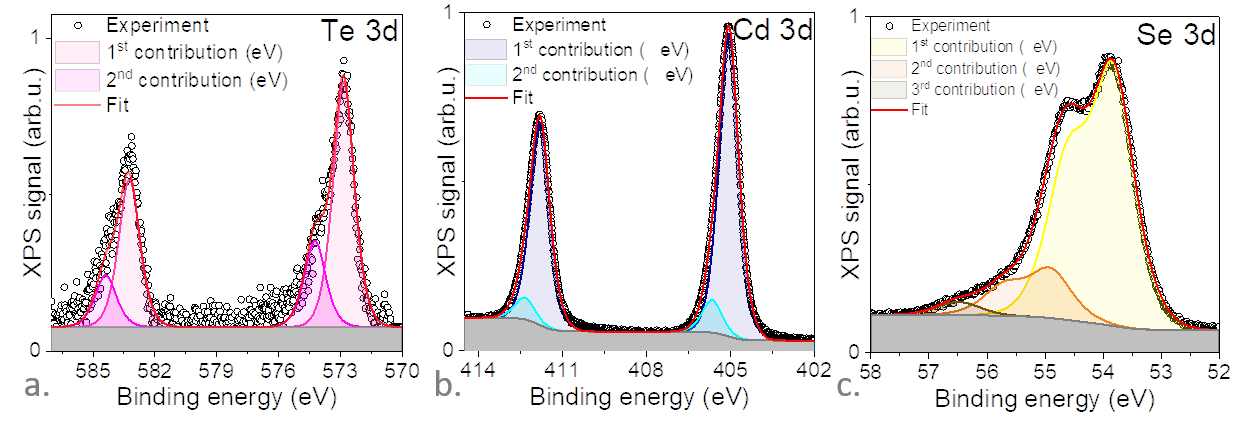
**

**Supplementary figure 10 Core-level analysis of the core/crown/crown NPL**. X-ray photoemission signal corresponding to a. Te 3d, b. Cd 3d and c. Se 3d core-level electrons.

The cut-off of the secondary electrons reveals a work function of 4.3 eV, see **Supplementary figure 11**a, while the valence band is located 1.2 eV below the Fermi level. For 4.5 ML CdSe NPL, the band edge energy is 2.4 eV, meaning that the Fermi level is close to the middle of the gap. Usually, for CdSe only the material is *n-*type only (*ie* Fermi level in the upper part of the band gap), thus this quasi intrinsic behavior observed here is clearly due to the presence of CdTe which nature is *p-*type.^3^

**
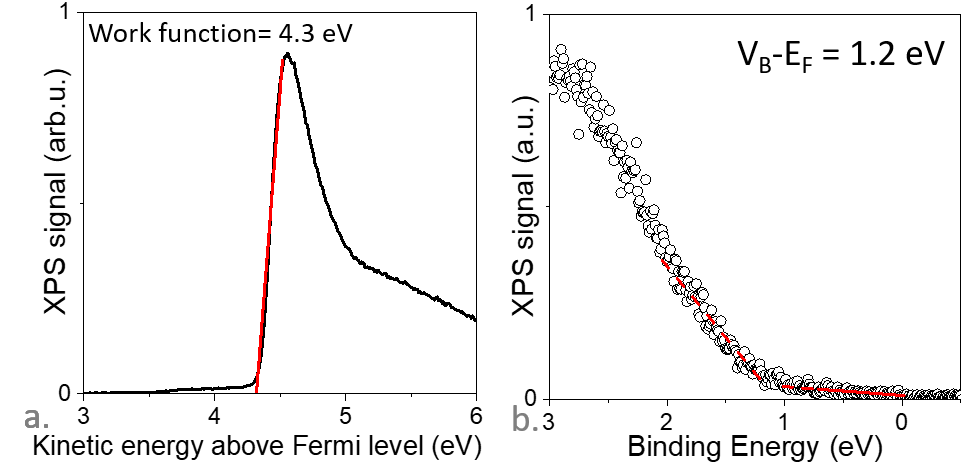
**

**Supplementary figure 11 Band alignment analysis by X-ray photoemission spectroscopy.** X-ray photoemission signal relative to (a.) secondary electrons cut-off and (right) and (b.) valence band for core/crown/crown NPL.

# Supplementary note 8: Evidence for coupling of the green and indirect exciton

If high photon energy (*ie* above CdSe band edge) is used to excite the system, both the green (direct exciton in CdSe) and red (indirect exciton at the CdSe/CdTe interface) PL signal can be excited, as shown in **Supplementary figure 12** and Figure 1. When lower photon energies (ie below CdSe band edge) are used, no green PL is obtained, however we still observe a red PL signal, see **Supplementary figure 12**.


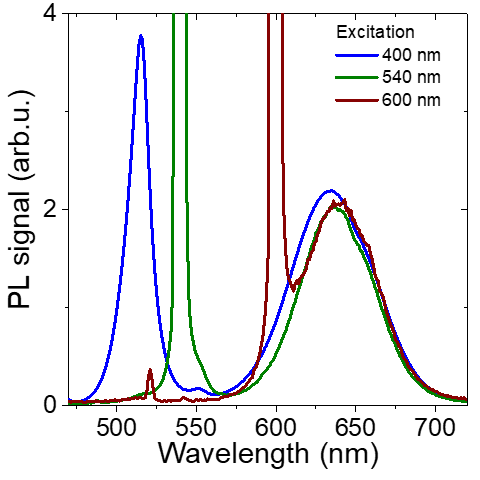


**Supplementary figure 12 PL spectra for various excitation wavelength** **of CdSe/CdTe/CdSe core/crown/crown NPL.**

In the main text (Figure 2b) we provide the TA signal while high photon energy is used. In **Supplementary figure 13**, we also have measured the TA signal, while excitation is below the absorption of both CdSe and CdTe (*ie* when only the indirect exciton can be exited). In this case, we observe a bleach of the probe signal if the latter is resonant with the indirect exciton, but also for the green signal. The latter is the signature of the photoexcited electron resulting from the indirect excitation and that is localized in CdSe. Its presence induces a bleach of the direct exciton signal in CdSe.


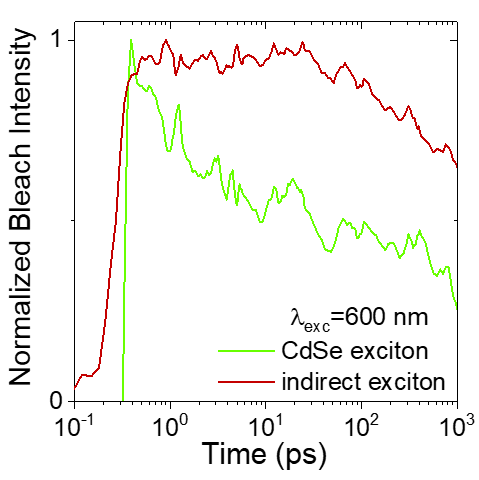


**Supplementary figure 13 Transient absorption signal for the green and red features**, while the NPLs are pumped at 600 nm (ie below CdSe excitation).

# Supplementary note 9: Origin of the two luminescence

- **Photoluminescence excitation Measurements**

An important question relative to the observation of 2 PL signal is the confirmation that they come from a single population of particle. To answer this question, we have performed photoluminescence excitation (PLE) measurements. PLE spectra for emission at the CdSe band edge and for emission matching the red indirect exciton are both overlapping with the absorption spectrum. We can thus conclude that both emission result from a single population of NPL.


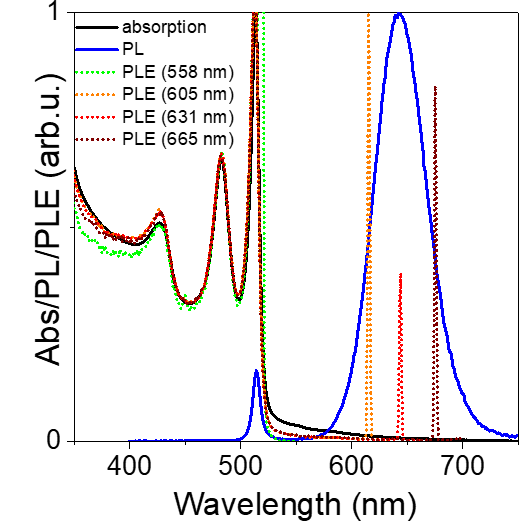


**Supplementary figure 14 PLE spectra at various excitation wavelengths for CdSe/CdTe/CdSe core/crown/crown NPL.** Absorption, PL spectrum (obtained using excitation at 350 nm) and PL spectra acquired for emission at 518, 605, 631 and 665 nm.

- **Single particle Measurements**

In order to validate the intrinsic origin of the bicolor emission, the PL response of highly diluted NPLs phases was investigated at cryogenic temperature (T ≈ 7 K). Dispersed ensembles were obtained on coverslips substrates by dilution of the original hexane solutions and a simple dropcasting method. **Supplementary figure 15**(a)-(c) shows the evolution of a typical PL spectrum as the dispersion increases ((a) is for a dilution by a factor 50 whereas in (b) and (c) the dilution factor reaches 10^3^). In (a) the structuration of the main X band (CdSe external crown exciton) clearly shows up while single objects reponses are evidenced in **Supplementary figure 15** (b) and (c). In all the experiments the X band is easily observable – at around 500 nm - due to the sharpness of the peaks, characteristic of a type-I exciton having a large oscillator.^4,5^ Compared to the PL spectra measured from the solution the bands are displaced towards the high energy side of the visible spectrum (Δλ ≈ 15-20 nm) in accordance with the predictions of the Varshny law for CdSe and CdTe materials (see Supporting Information of ref^5^). In **Supplementary figure 15** (c) the spectrometer resolution (300 lines/mm grating) provides an upper limit of the X emission linewidth (≈1 meV) while the IX emission band (CdSe/CdTe interface exciton) remains broad, developing in the 600 nm - 650 nm interval typically. The main point is that, in the course of the experiments, *the X and IX bands are observed concomitantly each time an individual particle is addressed*. This systematic observation of joint emissions is a conspicuous demonstration that the X and IX emission originate from the same single nanoparticle.

Even if not completely understood at present time, the IX ‘line’ is always much broader (nearly two orders of magnitude compared to the X line) and keeps a large spatial extension under the form of a more or less pronounced multi-peak progression despite the low temperature study conditions. This typical progression could be recently assigned to LO phonons replica in an analoguous CdSe/CdTe single-crown heterostructure, each component (among which the zero-phonon line) being itself broadened by spectral diffusion processes occuring on a timescale well below the observation/integration time of the experiment^5^. For clarity reasons, (i) the spectrum in **Supplementary figure 15** (c) is displayed with a 10 x magnification of the IX band in panel (d) and, (ii) a log-scale representation is also provided in **Supplementary figure 15** (e). Additional spectra associated to three other single NPLs responses are plotted in panels **Supplementary figure 15** (f) – (h).


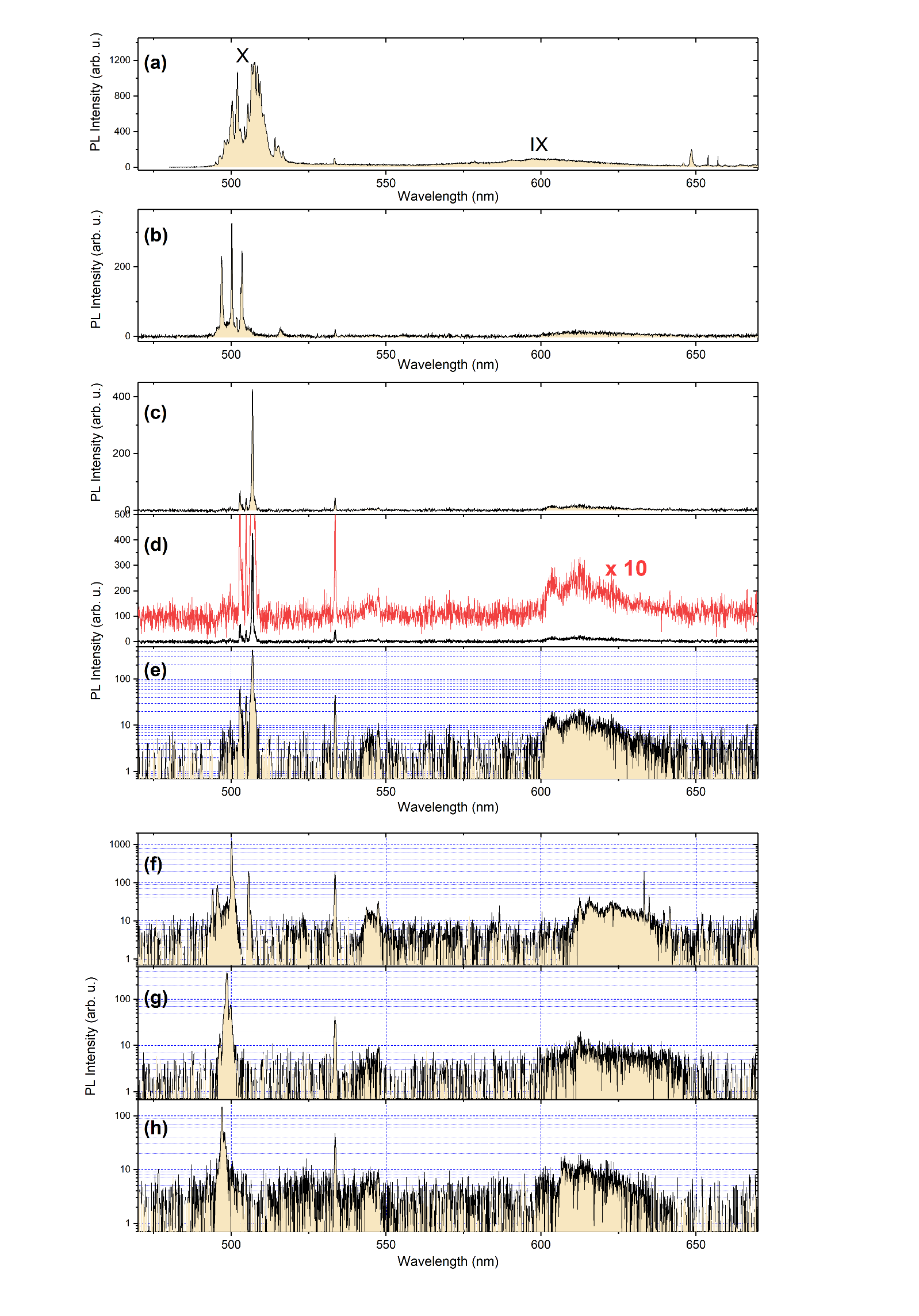


**Supplementary figure 15** **Optical spectroscopy of diluted phases**. Low temperature micro-photoluminescence spectra of double crown NPLs phases with increasing dilution from (a) (dilution factor ≈50) to (c) (dilution factor ≈10^3^). X is for the ‘direct’ exciton (or type-I CdSe external crown exciton) while IX is for the ‘indirect’ exciton (or type-II exciton). (d) Same as (c) but with a 10 x magnification of the IX emission band (red line). (e) Same as (c) with a log scale. (f)-(h) Three other characteristic single particle spectra. The line emerging at 532 nm is from the scattered light from the Ti-sapphire pump laser. The secondary structures around the main X line may be assigned to phonons replica or lower intensity emitting particles that are less efficiently coupled to light.

# Supplementary note 10: Electronic structure of the electron and hole ground state

To gain insight into the electronic structure of the band edge electrons, holes and excitons, we model our NPLs using effective mass Hamiltonians, including spatial and dielectric confinements, coupled with a self-consistent calculation to account for the electron-hole attraction (see Methods in the main text). We consider a cuboidal core-crown-crown (CdSe/CdTe/CdSe) NPL, as shown in the figure below (**Supplementary figure 16**).

**
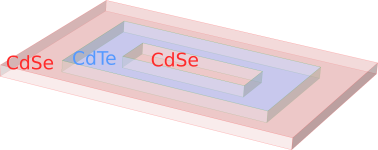
**

**Supplementary figure 16. Geometry and composition of the NPL under study.**

The dimensions we choose here correspond to the mean values inferred from TEM for the reference sample investigated in the main text. Thus, the thickness is 4.5 ML, the CdSe core measures ≈7x30 nm^2^ and the CdTe crown is 9x32 nm^2^ (that is, 2 extra nm per side with respect to the core). The external CdSe crown has variable dimensions, $L_{x}^{CdSe-crown} \times L_{y}^{CdSe-crown}$ nm^2^.


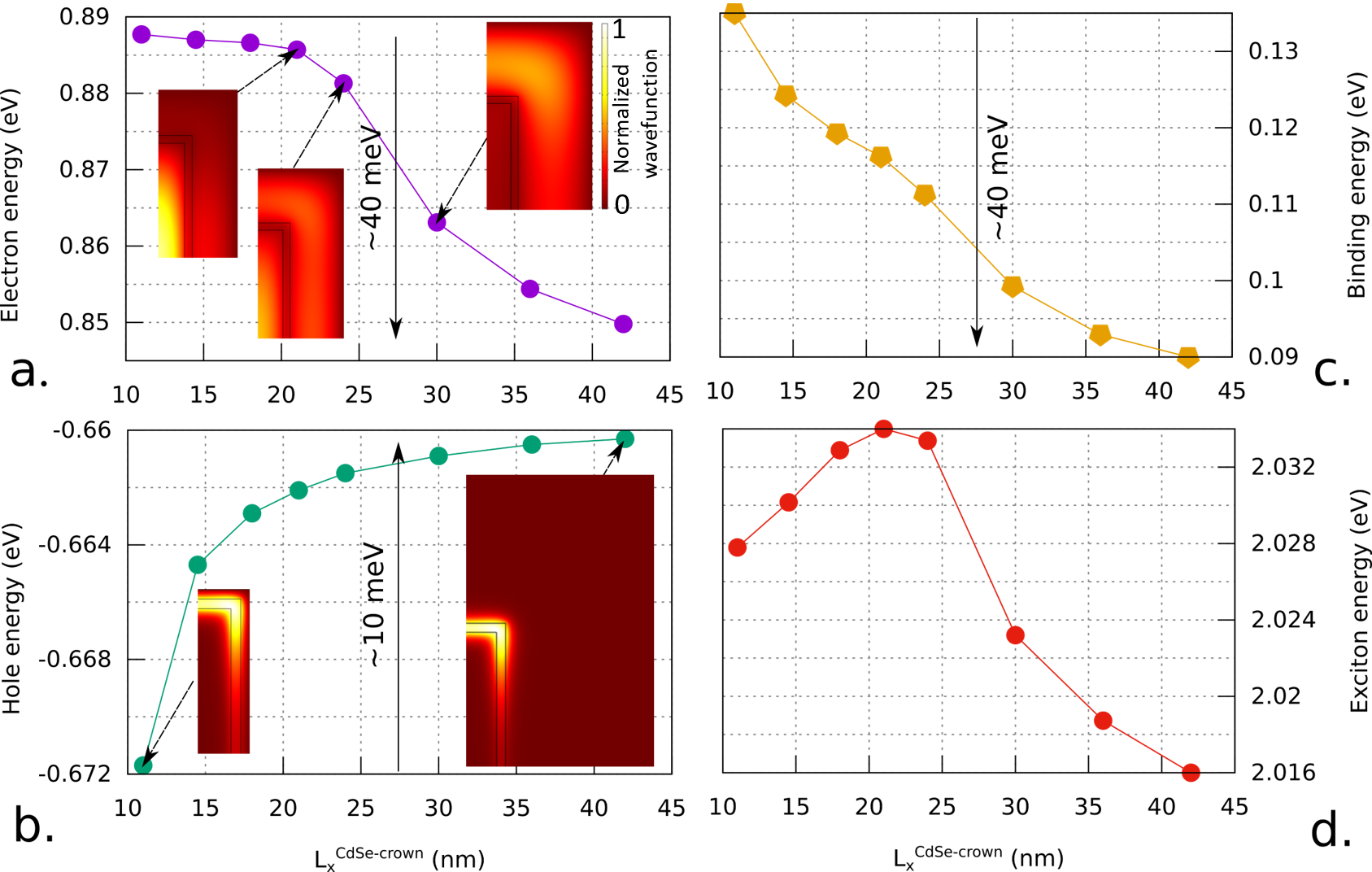


**Supplementary figure 17. Effect of the external CdSe crown on the electronic structure** of (a.) the electron, (b.) hole, (c.) exciton binding energy and (d.) exciton energy. The insets in (a.) and (b.) show the wavefunction localization (scale bar is the same as Figure 2c) for different dimensions (only one-quarter of the NPL is shown; the rest is symmetrical). In the calculations, the length ($L_{y}^{CdSe-crown}$) increases by the same amount as the width ($L_{x}^{CdSe-crown}$). In d, the band gap has been chosen to fit the experimental energy at $L_{x}^{CdSe-crown}$ (≈2.02 eV). It is the shift in the crown size that matters.

First, we investigate the influence of the external CdSe crown, which is a distinctive factor as compared to binary (CdSe/CdTe) heterostructures. The electron is quite insensitive to the external crown up to a width of $L_{x}^{CdSe-crown}$=20 nm. Around this point, quantum confinement in the CdSe crown becomes weaker than that in the core, so the electron moves outside and is energetically stabilized by a few tens of meV (**Supplementary figure 17**a). The top-most hole state becomes more stable as well (**Supplementary figure 17**b) because the CdSe crown provides weaker confinement as compared to the hard wall experienced in its absence. Since this is an indirect effect, the stabilization is smaller than for the electron (few meV). When electron-hole interactions are included, the ground state is significantly modified (see Fig.2c and 2d in the main text). This is partly related to the large binding energies (90-130 meV in **Supplementary figure 17**c). The total exciton energy (**Supplementary figure 17**d) experiences a moderate blueshift, followed by a redshift. This is a result of the balance between the weaker quantum confinement of electron and hole, and the weaker electron-hole attraction (binding energy). Altogether the exciton shift is of a few meV only. This value is much smaller than the bandwidth of the red peak in the emission spectra, which explains the absence of a clear shift in the experiments (Supplementary Note 4).

Next, we investigate the influence of the CdTe crown lateral size. The external CdSe crown is now set to 42x65 nm^2^ (a sufficiently large value not to interfere with the hole in CdTe), and the length $L_{y}^{CdTe-crown}$) and width ($L_{x}^{CdTe-crown}$) of the CdTe crown are varied. Increasing the CdTe crown dimensions stabilizes the hole ground state by reducing the lateral confinement, up to a few hundreds of meV (**Supplementary figure 18**a). This translates into a pronounced redshift in the exciton energy (**Supplementary figure 18**b), which is consistent with the PL redshift observed in Supplementary Note 3.


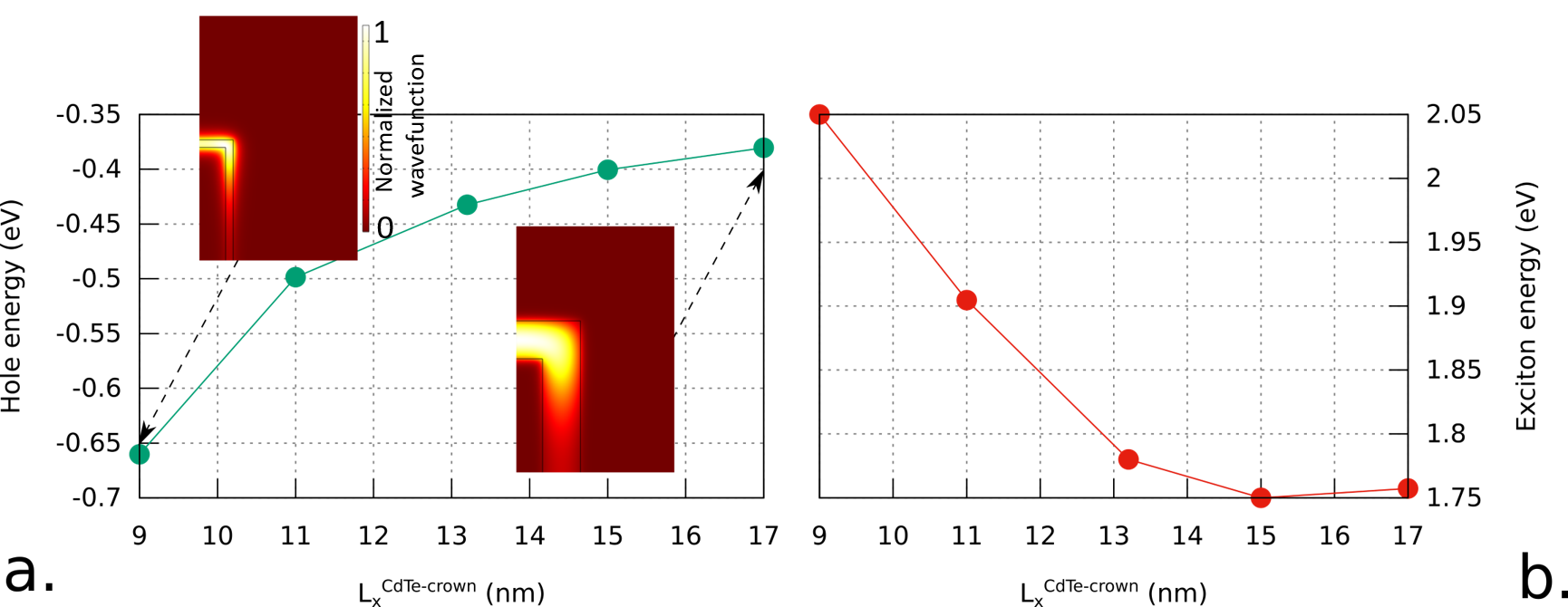


**Supplementary figure 18. Effect of the inner CdTe crown on the electronic structure** of (a.) hole, (b.) exciton energy. The insets in (a.) show the wave function localization (scale bar is the same as Figure 2c) for different dimensions. In the calculations, the length ($L_{y}^{CdTe-crown}$) increases by the same amount as the width ($L_{x}^{CdTe-crown}$).

# Supplementary note 11: Absence of hole blockade in the CdTe crown

The green-to-red emission ratio in CdSe/CdTe/CdSe NPLs increases with the incident power, as shown in Fig.4a of the main text. We ascribe this phenomenon to a greater effect of Auger recombination for the red feature as compared to the green one (see discussion in the main text). An alternative explanation for a similar observation (dual-color emission with power dependence) had been proposed in CdSe/CdS quantum dots^6^. Namely, it was the filling of the core with one hole which prevented further filling through a Coulomb blockade effect. To study if Coulomb blockade is also present in our NPLs, we calculate the ground state energy of few-interacting holes in NPLs. The NPLs have 4.5 ML thickness, a CdSe core of 10x30 nm^2^ and an external CdSe crown of 42x65 nm^2^. Two CdTe crown dimensions are considered for comparison, 13x33 nm^2^ (3 extra nm in each direction with respect to the core, similar to the reference sample of the main text) and 22x42 nm^2^ (12 extra nm).

We calculate the ground state of one to five interacting holes confined in the CdTe crown using configuration interaction routines, which duly account for Coulomb correlations (see Methods). **Supplementary figure 19** shows the hole-hole repulsion energy experienced by resident carriers. While the repulsion energy per hole increases steadily, it remains well below the band offset of CdSe/CdTe (690 meV). This suggests that the repulsions are not strong enough to produce a Coulomb blockade effect at least up to 5 holes.

**
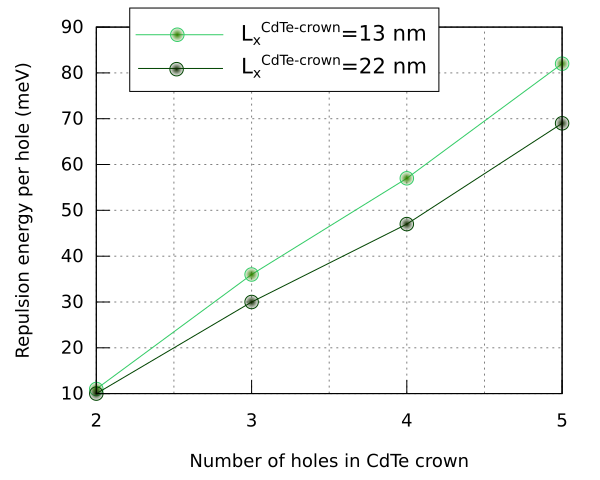
**

**Supplementary figure 19. Coulomb repulsion energy per hole** in the ground state of CdSe/CdTe/CdSe NPLs**.**

The same conclusion holds if one represents the addition energy (the equivalent of electron affinity for solid-state systems), which is calculated as $\Delta=E(N+1)-2E(N)+E(N-1)$. Here, $E(N)$is the ground state energy of *N* interacting holes. **Supplementary figure 20** shows that the addition energies present the usual saw-tooth structure expected for confined systems, with maxima for closed shells and minima for open shells^7^. There is no trace of low values expected when the next hole must be placed in the CdSe domain, neither for the larger nor for the smaller CdTe crown NPL.


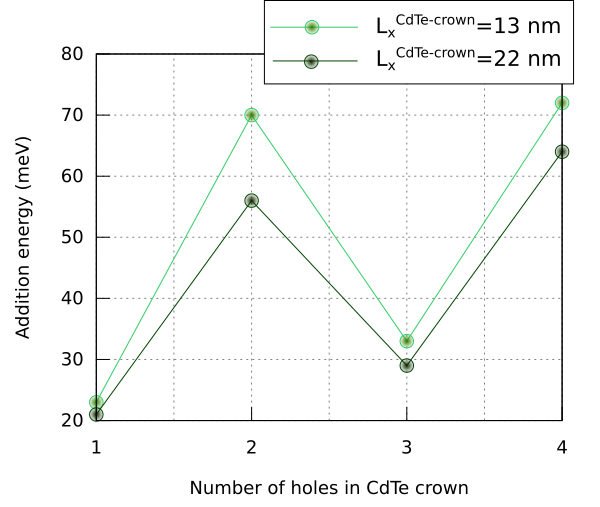


**Supplementary figure 20. Addition energies as a function of the number of holes** confined in CdSe/CdTe/CdSe NPLs.

We also compute the ionization energy (that is, the energy required to eject one hole from the CdTe crown to CdSe) in the NPL. It is calculated as $E_{\mathrm{ion}}=(E(N-1)+E_{\mathrm{cont}})-E(N)$. That is the energy difference between the *N*-hole ground state and the *N-1* hole ground state with one hole in the first CdSe quasi-continuum state (neglecting its Coulomb interaction with the confined holes). An example of the ionization process is illustrated in the schematics of **Supplementary figure 21**a.

**
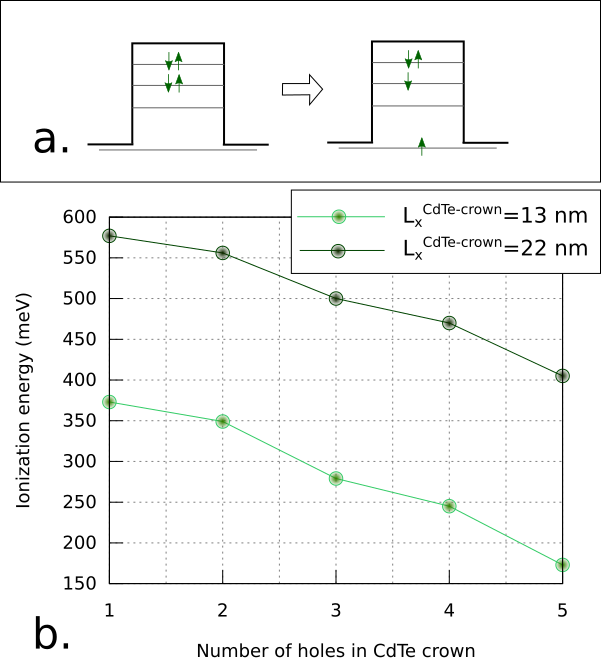
**

**Supplementary figure 21. Ionization energies** as a function of the number of holes confined in CdSe/CdTe/CdSe NPLs. a. Schematic of an ionization process. b. Calculated ionization energies.

**Supplementary figure 21**b shows that up to 5 holes, the energy needed to remove one hole from the CdTe crown is still over 150 meV, well above the thermal energy at room temperature.

All the above results point to NPLs showing a distinct behavior as compared to CdSe/CdS quantum dots. Despite the strong Coulomb repulsions (enhanced by dielectric confinement), the large in-plane dimensions and the large CdTe/CdSe valence band offset enable the CdTe crown to host several holes.

# Supplementary note 12: Stimulated emission and multiexciton emission

Under fs pulse excitation, we can observe amplified spontaneous emission (ASE) from a thin film of CdSe/CdTe/CdSe core/crown/crown NPL, see **Supplementary figure 22**a. Note that ASE is only observed from the CdSe area of the NPL. The threshold is 42 µJ.cm^-2^ (**Supplementary figure 22**b) which is above the best-reported values (from 4.5 ML CdSe). This can be directly connected to the charge transfer process which diminishes population inversion and re-absorption losses from the CdTe and charges transfer absorption bands^8–11^.


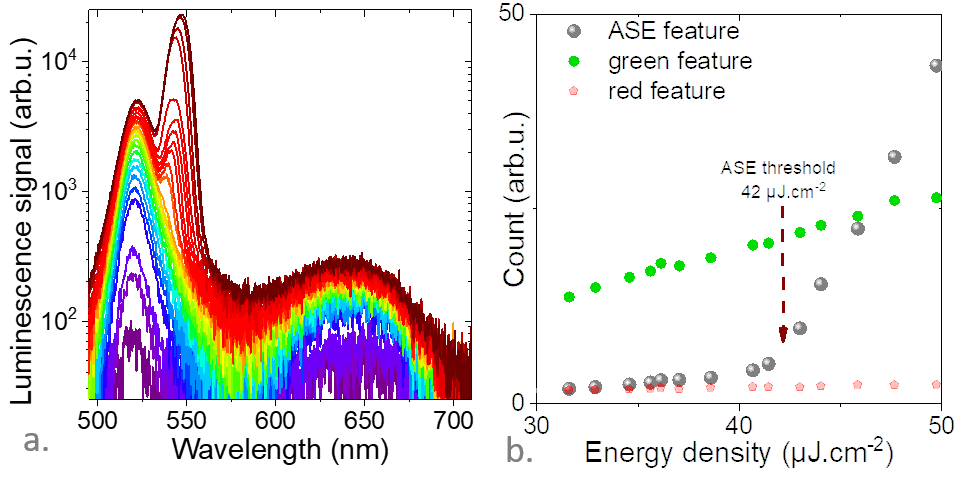


**Supplementary figure 22 Stimulated emission.** a. Luminescence spectra for a thin film made of CdSe/CdTe/CdSe core/crown/crown NPL under various incident powers. b. Magnitude of the red, green and stimulated green emissions as a function of the incident energy density.

**Supplementary figure 23** shows the gain spectrum. The relative magnitude of gain at the green exciton is clearly larger than the one observed for the indirect transition. However, as the inset shows, the gain is still existing at wavelengths matching the red exciton.


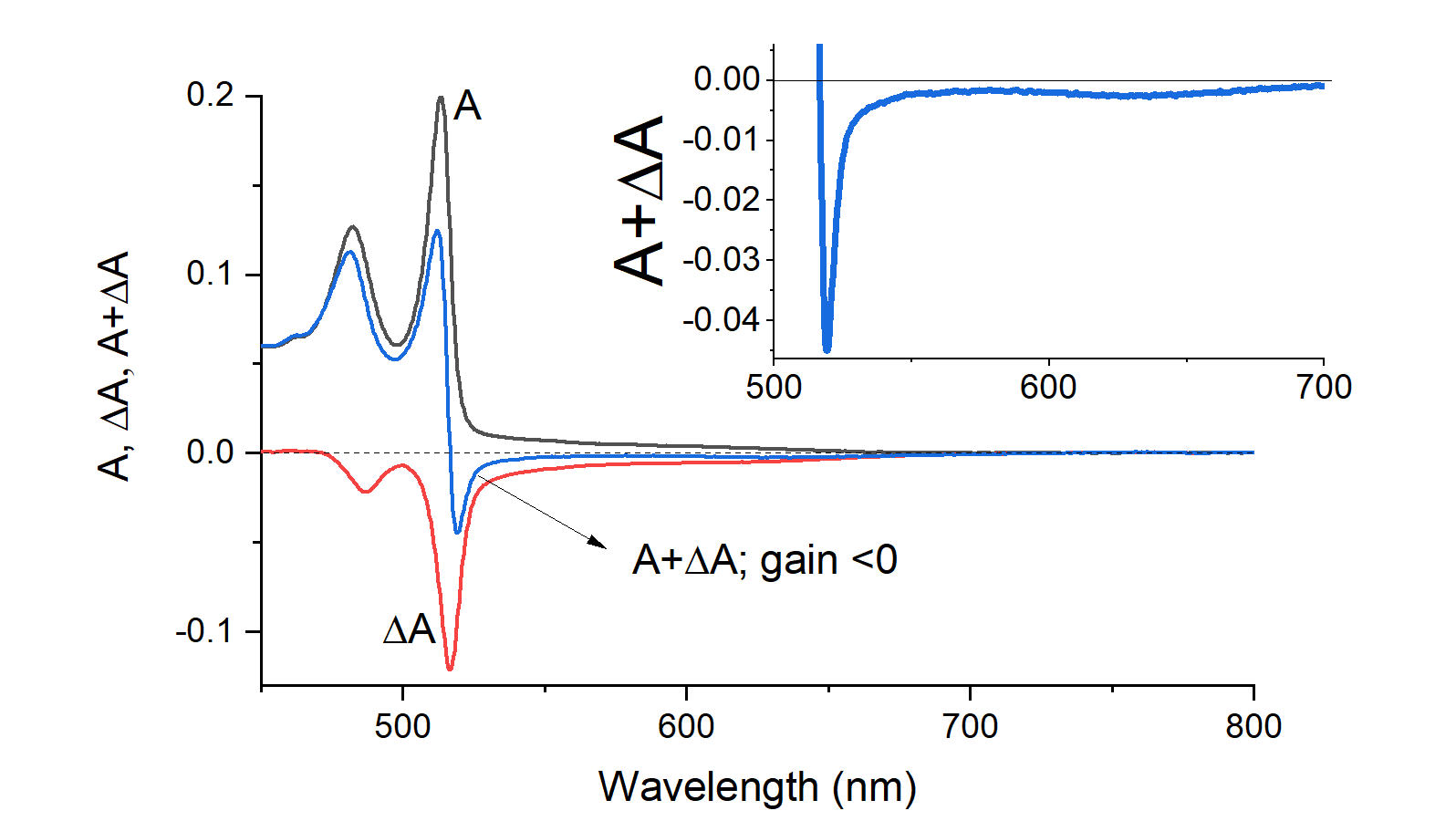


**Supplementary figure 23 Gain spectrum**. Plot overlaying the absorption (black) of a CdSe/CdTe/CdSe NPL sample with the transient spectrum at 3 ps pump-probe delay (ΔA, red), and the gain spectrum (A+ΔA, blue). Regions for which A+ΔA<0 represent optical gain. The inset shows gain at the green band and weaker, but still detectable, gain arising from the charge transfer feature in the red. This indicates that stimulated emission from both features is possible, although the modal gain is much larger for the green feature.

Under strong excitation, we observe a shift and a broadening of the two PL peaks. In particular, the green emission experiences a redshift, attributed to the radiative biexciton occurring for fluence above 20 µJ.cm^-2^, which is close to the threshold where ASE is observed 42 µJ.cm^-2^. This suggests that the ASE mechanism in this core/crown/crown NPL is the same as for CdSe NPL.


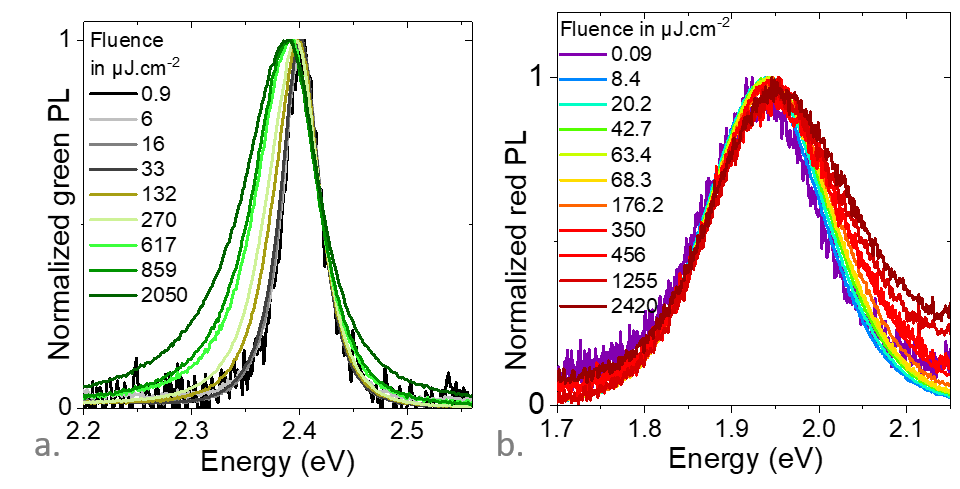


**Supplementary figure 24. Evidence for biexciton emission** a. and b. Normalized photoluminescence intensity at (a) the green emission feature and (b) red emission feature at several fluences. At high fluence, the green feature in (a) shows a red-shift due to an attractive biexciton. Under the same conditions, the red feature in (b) shows a blue-shift typical of a type II, repulsive biexciton.

# Supplementary note 13: Effect of geometrical factor on the power threshold relative to green emission prevalence

**Supplementary figure 25** shows the power-dependent spectrum of CdSe/CdTe/CdSe core/crown/crown NPL for which the CdTe crown is typically four times larger than the one studied in the main text.


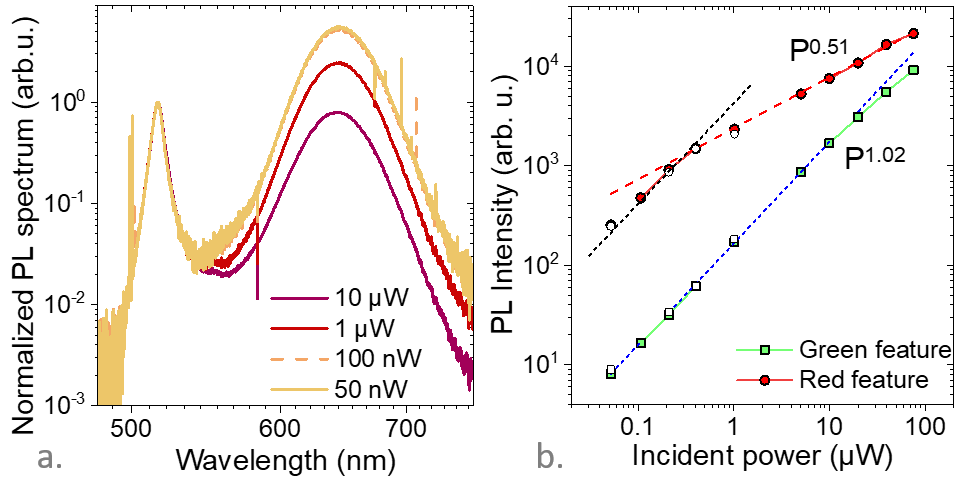


**Supplementary figure 25 Power dependence of luminescence in presence of a large CdTe crown**. a. Luminescence spectra, normalized by the green peak under various incident powers. b. Magnitude of the green (square) and red peaks (circle) as a function of the incident power. Dashed lines are power law fit.

# Supplementary note 14: LED characterization

The LED based on CdSe/CdTe/CdSe core/crown/crown NPL as emissive material presents a low turn-on voltage (1.65 V, see **Supplementary figure 26**a) corresponding to a sub band gap (*ie* even considering the type II interface) operation. External quantum efficiency (EQE) is modest (10^-2^ %, see **Supplementary figure 26**b) and this must be connected to the large drop of PL efficiency resulting from the NPL integration into the LED stack. The maximum of luminance is observed at around 100 Cd.m^-2^ which still matches the need for displays, but is clearly insufficient for lightning.


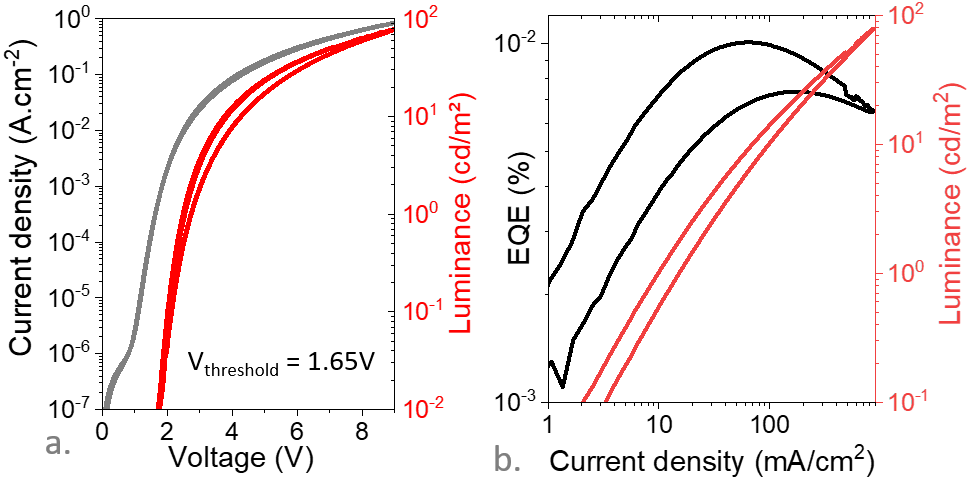


**Supplementary figure 26 LED characterization**. a. Current density (black) and luminance (red) according to the applied bias in a LED based on core/crown/crown NPL. b. External quantum efficiency (EQE in black) and luminance (red) according to the driving current for the same LED.

# Supplementary references

1. Ithurria, S. & Dubertret, B. Quasi 2D Colloidal CdSe Platelets with Thicknesses Controlled at the Atomic Level. *J. Am. Chem. Soc.* **130**, 16504–16505 (2008).

2. Carbone, L. *et al.* Synthesis and Micrometer-Scale Assembly of Colloidal CdSe/CdS Nanorods Prepared by a Seeded Growth Approach. *Nano Lett.* **7**, 2942–2950 (2007).

3. Lhuillier, E. *et al.* Investigating the *n*- and *p*-Type Electrolytic Charging of Colloidal Nanoplatelets. *J. Phys. Chem. C* **119**, 21795–21799 (2015).

4. Dufour, M. *et al.* Engineering Bicolor Emission in 2D Core/Crown CdSe/CdSe_1–x_Te_x_ Nanoplatelet Heterostructures Using Band-Offset Tuning. *J. Phys. Chem. C* **121**, 24816–24823 (2017).

5. Steinmetz, V. *et al.* Emission State Structure and Linewidth Broadening Mechanisms in Type-II CdSe/CdTe Core–Crown Nanoplatelets: A Combined Theoretical–Single Nanocrystal Optical Study. *J. Phys. Chem. C* **124**, 17352–17363 (2020).

6. Galland, C. *et al.* Dynamic Hole Blockade Yields Two-Color Quantum and Classical Light from Dot-in-Bulk Nanocrystals. *Nano Lett.* **13**, 321–328 (2013).

7. Tarucha, S., Austing, D. G., Honda, T., van der Hage, R. J. & Kouwenhoven, L. P. Shell Filling and Spin Effects in a Few Electron Quantum Dot. *Phys. Rev. Lett.* **77**, 3613–3616 (1996).

8. Kelestemur, Y. *et al.* Colloidal CdSe Quantum Wells with Graded Shell Composition for Low-Threshold Amplified Spontaneous Emission and Highly Efficient Electroluminescence. *ACS Nano* **13**, 13899–13909 (2019).

9. Guzelturk, B., Kelestemur, Y., Olutas, M., Delikanli, S. & Demir, H. V. Amplified Spontaneous Emission and Lasing in Colloidal Nanoplatelets. *ACS Nano* **8**, 6599–6605 (2014).

10. She, C. *et al.* Red, Yellow, Green, and Blue Amplified Spontaneous Emission and Lasing Using Colloidal CdSe Nanoplatelets. *ACS Nano* **9**, 9475–9485 (2015).

11. Zhang, L. *et al.* Low-Threshold Amplified Spontaneous Emission and Lasing from Thick-Shell CdSe/CdS Core/Shell Nanoplatelets Enabled by High-Temperature Growth. *Adv. Opt. Mater.* **8**, 1901615 (2020).
